# Gene mutations and increased levels of p53 protein in human squamous cell carcinomas and their cell lines.

**DOI:** 10.1038/bjc.1993.238

**Published:** 1993-06

**Authors:** J. E. Burns, M. C. Baird, L. J. Clark, P. A. Burns, K. Edington, C. Chapman, R. Mitchell, G. Robertson, D. Soutar, E. K. Parkinson

**Affiliations:** Cancer Research Campaign, Beatson Institute for Cancer Research, Garscube Estate, Bearsden, Glasgow, UK.

## Abstract

**Images:**


Br. J. Cancer (1993), 67, 1274–1284 © Macmillan Press Ltd., 1993

### Gene mutations and increased levels of p53 protein in human squamous cell carcinomas and their cell lines

J.E. Burns[1], M.C. Baird[1], L.J. Clark[1], P.A. Burns[1], K. Edington[1], C. Chapman[1], R. Mitchell[2], G. Robertson[3], D. Soutar[3] & E.K. Parkinson[1]

[1]*Cancer Research Campaign, Beatson Institute for Cancer Research, Garscube Estate, Switchback Road, Bearsden, Glasgow, G61 1BD;* [2]*Department of Oral and Maxillofacial Surgery, University of Edinburgh, Old High School Yards, Edinburgh;* [3]*Canniesburn Hospital, Switchback Road, Bearsden, Glasgow, UK.*

**Summary** Using immunocytochemical and Western blotting techniques we have demonstrated the presence of abnormally high levels of p53 protein in 8/24 (33%) of human squamous cell carcinomas (SCC) and 9/18 (50%) of SCC cell lines. There was a correlation between the immunocytochemical results obtained with eight SCC samples and their corresponding cell lines.

Direct sequencing of PCR-amplified, reverse transcribed, p53 mRNA confirmed the expression of point mutations in six of the positive cell lines and detected in-frame deletions in two others. We also detected two stop mutations and three out-of-frame deletions in five lines which did not express elevated levels of p53 protein. Several of the mutations found in SCC of the tongue (3/7) were in a region (codons 144–166) previously identified as being a p53 mutational hot spot in non-small cell lung tumours (Mitsudomi *et al.*, 1992). In 11/13 cases only the mutant alleles were expressed suggesting loss or reduced expression of the wild type alleles in these cases. Six of the mutations were also detected in the SCCs from which the lines were derived, strongly suggesting that the mutations occurred, and were selected, *in vivo*.

The 12th mutation GTG→GGG (valine→glycine) at codon 216 was expressed in line SCC-12 clone B along with an apparently normal p53 allele and is to our knowledge a novel mutation. Line BICR-19 also expressed a normal p53 allele in addition to one where exon 10 was deleted.

Additionally 15 of the SCC lines (including all of those which did not show elevated p53 protein levels) were screened for the presence of human papillomavirus types 16 and 18 and were found to be negative.

These results are discussed in relation to the pathogenesis of SCC and the immortalisation of human keratinocytes *in vitro*.

In recent years several lines of evidence have characterised p53 as a tumour suppressor gene. There is a frequent loss of heterozygosity at the p53 locus in many types of human tumour which might suggest a reduction to homozygosity of mutated, deleted or rearranged p53 alleles in these cases (Fearon *et al.*, 1987; Yokota *et al.*, 1987; Vogelstein *et al.*, 1988; Baker *et al.*, 1989; Weston *et al.*, 1989). Furthermore, alterations within the coding sequence of the p53 gene are commonly observed in human cancers (Nigro *et al.*, 1989, see Hollstein *et al.*, 1991; Caron de Fromentel & Soussi, 1992 for reviews) including squamous cell carcinomas (SCC) of the lung (Chiba *et al.*, 1990) oesophagus (Hollstein *et al.*, 1990) anus (Crook *et al.*, 1991*b*), larynx (Maestro *et al.*, 1992), oral cavity (Brachman *et al.*, 1992) and epidermis (Brash *et al.*, 1991, Pierceall *et al.*, 1991). Additionally, it is known that many mis-sense p53 mutations induce changes of a probable conformational nature, which increase the half-life of the protein from 20 m (Oren *et al.*, 1981) to several h (Finlay *et al.*, 1988). As a consequence of this the p53 protein is rendered unusually detectable by conventional Western blotting and immunocytochemical techniques. Significantly, increased p53 protein levels have also been reported in a wide range of human tumours (Cattoretti *et al.*, 1988, van der Berg *et al.*, 1989, Bartek *et al.*, 1990*a,b*, Iggo *et al.*, 1990, Rodrigues *et al.*, 1990) including SCC (Field *et al.*, 1991, Bennett *et al.*, 1991, Gusterson *et al.*, 1991; Maestro *et al.*, 1992). The case for p53 as a suppressor gene is further strengthened by the examples of Li-Fraumeni Syndrome (LFS) patients who are predisposed to several cancer types (Li & Fraumeni 1969) and also carry germ-line p53 mutations (Malkin *et al.*, 1990, Srivastava *et al.*, 1990, Law *et al.*, 1991). Some of these LFS patients have also been shown to be heterozygous for the p53 mutation in the somatic tissue but homozygous for the same mutation in the tumour tissue (Malkin *et al.*, 1990). Furthermore, fibroblasts from LFS individuals are predisposed to spontaneous *in vitro* immortalisation and neoplastic progression (Bischoff *et al.*, 1990, 1991).

Experimental evidence to support p53 as a tumour suppressor comes from the observation that the wild type p53 gene, but not certain mutants, can exert a reversible (Michalovitz *et al.*, 1990) growth suppressive effects. Furthermore, this inhibition of cell proliferation by p53 is specific to transformed cells which harbour an altered p53 gene (Finlay *et al.*, 1989) including those derived from human tumours (Baker *et al.*, 1990, Mercer *et al.*, 1990; Diller *et al.*, 1990; Casey *et al.*, 1991). In other instances, the wild type p53 gene can suppress human tumour formation from xenografts (Chen *et al.*, 1990; 1991, Cheng *et al.*, 1992). It has also been shown that mice which are homozygous for a null p53 mutation are prone to early spontaneous tumour development, indicating that *loss* of p53 function is enough to contribute to tumour development (Donehower *et al.*, 1992).

Further support for viewing p53 protein as a tumour suppressor comes from the observations that it is bound (and presumed to be inactivated) by SV40 large T (Lane & Crawford, 1979; Linzer & Levine, 1979), adenovirus 5 EIB (Sarnow *et al.*, 1982) and human papillomavirus (HPV) E6 proteins (Werness *et al.*, 1990; Crook *et al.*, 1991*a*), and may even be inactivated by degradation in the case of HPV16 and HPV18 (Scheffner *et al.*, 1990; Crook *et al.*, 1991*a*). The last two viruses are of particular significance to our study since they have been reported to occur in both normal and malignant tissue of the oral cavity (Maitland *et al.*, 1987; 1989, Yeudall & Campo, 1991). In addition, transfection of these viruses *in vitro* into human keratinocytes from both the epidermis (Pirisi *et al.*, 1987; Kaur & McDougall, 1988) and the oral cavity (Park *et al.*, 1991) leads to their immortalisation, and both the E6 and E7 proteins are important for this process (Hawley-Nelson *et al.*, 1989; Munger *et al.*, 1989). Such immortalised keratinocytes readily progress to malignancy (Hurlin *et al.*, 1991).

In addition to the evidence supporting a role for p53 as a tumour suppressor gene there is also evidence that some 'gain of function' p53 mutations (Halevy *et al.*, 1990) can give the cells which possess them a growth advantage even in the

Correspondence: E.K. Parkinson.
Received 6 August 1992; and in revised form 21 January 1993.

absence of wild type p53 protein (Wolf *et al.*, 1984; Chen *et al.*, 1990). Therefore, if the role of p53 mutations in the pathogenesis of SCC is to be understood, the availability of well characterised cell lines carrying p53 alterations would make an important contribution, particularly if the same p53 alterations could be detected in the tumours from which the cell lines were derived (see Sakai & Tsuchida 1992). In this article we report the frequent detection of p53 mutations and elevated levels of protein in SCC of the epidermis and oral cavity as well as in a series of SCC cell lines which are shown to lack detectable HPV16 and 18 DNA. In six of the cell lines we also show that the p53 mutations present *in vitro* are also present *in vivo*.

### Materials and methods

### *Cell culture and SCC cell lines*

Human epidermal keratinocytes were prepared from infant foreskin samples as described (Parkinson *et al.*, 1986) and grown in Dulbecco's modified Eagle medium (DMEM) containing 20% foetal bovine serum (a selected lot), 0.4 $\mu g\ ml^{-1}$ hydrocortisone (Rheinwald & Green, 1975) and 10 $ng\ ml^{-1}$ cholera toxin (Green, 1978) in the presence of lethally irradiated Swiss 3T3 fibroblasts (Rheinwald & Green, 1975). Human foetal fibroblasts were prepared by collagenase digestion of whole skin fragments and were cultured in the same medium. All of the SCC lines were cultivated in the presence of lethally irradiated 3T3 cells in DMEM, containing the optimum concentration of foetal bovine serum and 0.4 $\mu g\ ml^{-1}$ hydrocortisone. Lines SCC-4 to SCC-27, BICR-3, 6, 10, 16 and 19 grew optimally in 10% v/v foetal bovine serum, BICR-7 in 5% v/v foetal bovine serum and BICR-18 and BICR-22 in 2% foetal bovine serum. The properties of the SCC lines are listed in Table II. The squamous origin of the lines was confirmed by electron microscopy to reveal desmosomes and stratification and by the immunocytochemical detection of cytokeratins and involucrin (Rheinwald & Beckett 1981; Edington *et al.* – manuscript in preparation).

### *Tumour collection and TNM staging*

Surgically removed SCC of the head and neck region were bisected and placed either into culture medium for the derivation of cell lines or snap frozen to obtain sections for histology and/or immunocytochemistry. The tumours were staged using the UICC TNM convention (UICC, 1987). All the tumours described were confirmed as being SCC by the Pathology Department, Glasgow Royal Infirmary and all biopsies contained SCC material.

### *Immunocytochemistry*

Cells were grown on chamber slides (LabTek, Nunc) prior to rinsing with calcium and magnesium-free phosphate buffered saline (CMF-PBS) and fixation. Both cryostat tissue sections and cells were fixed for 20 min in 100% methanol on ice prechilled to – 20°C overnight (Gusterson *et al.*, 1991) followed by air drying for 40 min. Endogenous peroxidase activity was quenched by incubating the samples at room temperature for 10 min in 3% v/v hydrogen peroxide (Sigma) in methanol. The samples were then rinsed twice (10 min each) in CMF-PBS pH 7.4 prior to blocking for 30 min with 10% normal goat serum (Vectastain, Peterborough, UK). The specimens were then incubated for 1 h at room temperature with the mouse monoclonal antibody pl801 (Cambridge Bioscience, UK), which is known to recognise both mutant and wild type proteins (Banks *et al.*, 1986), at 3 $\mu g\ ml^{-1}$ in CMF-PBS containing 0.1% crystalline bovine serum albumin. The antigens were then visualised by treatment of the specimens with biotinylated second antibody followed by streptavidin as supplied in the Vectastain ABC kit (Vectastain, Peterborough, UK).

After the final rinse in CMF-PBS pH 7.6 containing 0.15 M NaCl and 0.05% Tween 80 the cells or sections were incubated with 0.6 $mg\ ml^{-1}$ diaminobenzidine (Sigma) and 0.06% hydrogen peroxide (Sigma) in CMF-PBS pH 7.4 for 7–8 min. The specimens were then rinsed in distilled water, mounted in Aquamount (BDH Chemicals, Poole U.K.) and sealed in nail varnish. Alternatively, some tumour sections were stained with haematoxylin and eosin, dehydrated, cleared and mounted in DPX mounting medium (BDH Chemicals). The specimens were photographed with Panotomic X film under phase contrast optics or bright field optics. A green filter was used for the immunoperoxidase samples photographed without counterstain. No staining was ever seen when the first antibody was omitted.

### *Western blotting*

Cells were harvested from subconfluent (50–80%) plates using trypsin and EDTA, pelleted through serum-containing medium, rinsed twice with CMF-PBS and extracted by the method of Scheffner *et al.* (1991). Proteins were measured using a Bio-Rad protein determination reagent using bovine serum albumin as a standard and the samples (50 $\mu g$/lane) were separated alongside Rainbow marker proteins on a 15% SDS polycrylamide gel prior to transfer to a nylon filter (Hybond C Super, Amersham) using the Bio-Rad semi-dry blotter. After washing in Tris-buffered saline (TBS) pH 8.0 the filters were stained in Ponceau's solution (Sigma) for 5 min and destained for 5 min in 5% v/v acetic acid to check the consistency of transfer and loading. The filter was blocked at 4°C overnight with staining in TBS and 0.1% Tween 20 (TBST) containing 5% v/v milk fat protein (TBSTM). Following this the filter was incubated at room temperature with pl801 antibody (1 $\mu g\ ml^{-1}$) in TBSTM, rinsed in TBSTM, and incubated with peroxidase-conjugated anti-mouse immunoglobulin (Amersham). After further rinsing the filter was dipped in luminol solution (ECL, Amersham) for one minute and exposed to Kodak X-Omat fast film for 5 min prior to development. Human epidermal keratinocytes and fibroblasts were used as controls for normal p53 levels, JW-2 as a p53 null control and HT29 as a positive control. All films were exposed so that the HT29 positive control produced the same intensity of signal in each blot.

### *Nucleic acid isolation*

RNA was extracted from exponentially growing cells using RNAzol B (Cinna/Biotecx), subjected to two ethanol precipitations and stored in aqueous solution at – 70°C. Genomic DNA was isolated directly from tumour cryostat sections by lysis of individual sections with 100 $\mu l$ of lysis buffer containing 10 m Tris-HCl, 10 mM EDTA, 10 mM NaCl, 4% N-lauryl sarcosine and 2.75 $mg\ ml^{-1}$ proteinase K, followed by overnight digestion at 37°C and EtOH precipitation. The resulting pellet was washed with 80% EtOH, dried, resuspended in 60 $\mu l$ TE and stored at 4°C. For PCR amplification this DNA was diluted 1/10 with TE. Genomic DNA was also isolated from cell lines by lysis with 4 M guanidinium thiocyanate followed by differential centrifugation through CsCl solutions, proteinase K digestion, then phenol extracted and EtOH precipitated. This DNA was stored in aqueous solution at 4°C and used to check the mutations found in mRNA in some cases.

### *Primers*

PCR and sequencing primers A, B, D, E and G were those described by Rodrigues *et al.* (1990). Downstream PCR primers E and G were 5′ biotinylated during synthesis.

For PCR amplification and sequencing of DNA the primers described by Brash *et al.* (1991) were used, the downstream primer of each pair being 5′ biotinylated during synthesis (see Figure 6).

The full list of primers was, as follows:

A: 5′CAGCTCCTACACCGGCGGCCCCTGCACCAG3′

B: 5'CCTGTCCCTTCCCAGAAAACC3'
D: 5'TAGTGTGGTGGTGCCCTATGAGCCG3'
E*: 5'biotin-GAGCCAACCTCAGGCGGCTCTCATAGGG CACC3'
G*: 5'biotin-GTGGGAGGCTGTCAGTGGGGAACAA3'
K: 5'CTACAAGCAGTCACAGCACAT3'
L: 5'AGAAGAAACCACGGATGGAG3'
Brash2U: 5'ACTGCCTTCCGGGTCACTGC3'
MA1: 5'TCCACGACGGTGACACGCTT3'
CC2*: 5'biotin-AAGGGACAGAAGATGACAGG3'
Brash5U: 5'TTCCTCTTCCTGCAGTACTC3'
Brash5D* 5'biotin-GCCCCAGCTGCTCACCATCG3'
Brash6U: 5'CTGATTGCTCTTAGGTCTGG3'
Brash6d*: 5'biotin-AGTTGCAAACCAGACCTCAG3'
Brash8U: 5'AGTGGTAATCTACTGGGACG3'
Brash9D*: 5'biotin-ATTCTCCATCCAGTGGTTTC3'

The primers for the amplification of HPV-16 and HPV-18 have been described previously (Yeudall & Campo, 1991) and amplify fragments of the E6/E7 (HPV-16) or E6 regions (HPV-18) which are 165 and 99 bp respectively.

The HPV16 primers were as follows:
5'TTAATTAGGTGTATTAACTG3'
5'TGCATGATTACAGCRGGGTT3'

The HPV18 primers were as follows:
5'ATCTGTGTGCACGGAACTAAC3'
5'AATGCAAATTCAAATACCTC3'

The HPRT primers were as follows:
Primer 1 5'CTTGCTGGTGAAAAGGACCC3'
Primer 2 5'GTCAAGGGCATATCCTACAA3'

These primers produce a PCR product of 275 bp.

SiHa DNA (Single copy HPV-16), W12 (HPV-16) and HeLa (HPV-18) were used as positive controls (see Pater & Pater, 1985) and each PCR reaction was carried out at least five times.

*PCR amplification*

For p53 amplification first strand cDNA was synthesised from 1 µg of total cellular RNA using a Perkin-Elmer Cetus RNA PCR kit, using random hexamers as primers. After the addition of Taq polymerase and 0.15 µM each PCR primer, amplification proceeded for 35 cycles of 95°C for 1 min and 60°C for 1 min + 2 s extension per cycle. Reaction conditions were as specified by the manufacturer.

Genomic DNA was amplified using a Perkin-Elmer Cetus PCR kit using the following conditions:

Exon 5: Buffer J (Brash *et al.* 1991)
Exon 6: Buffer J (Brash *et al.* 1991)
Exons 8/9: Buffer supplied with PCR kit

Amplification was carried out for 35 cycles of 94°C for 30 seconds, 55°C for 1 min, 72°C for 1 min.

For HPV-16 and HPV-18 amplification 0.5 µg genomic DNA and 2.5 U Taq DNA polymerase were used in a total reaction volume of 50 µl. Final concentration of other reagents were 200 µM dATP, dTTP, dGTP and dCTP; 10 mM Tris-HCl (pH 6.3); 50 mM KCl; 1.5 mM $MgCl_2$, 0.001% gelatin, 0.8 µM HPV primers and 40 mM HPRT primers.

The conditions were as follows 94°C prior to enzyme addition for 10 min, then 30 cycles of 94°C 1 min, 51°C 1 min, 72°C 1 min followed by 10 min at 72°C.

Fifteen µl of each reaction product were run on a 6% polyacrylamide gel along with a φX marker ladder. The gel was stained with ethidium bromide and photographed.

*Sequencing*

Single-stranded PCR products were purified using Dynal M-280 Streptavidin beads (Dynal UK Ltd) and stored at − 20°C in aqueous solution. Both biotinylated and non-biotinylated strands were isolated but only biotinylated products were found to give satisfactory sequencing results routinely.

Direct sequencing of single-stranded PCR products was carried out by the dideoxy chain termination method using a Sequenase 2.0 kit (United States Biochemical). Reaction products were analysed in 8% polyacrylamide gels. At least two independent PCR products were sequenced in each case. In the case of six BICR samples both cell line and tumour material was sequenced.

### Results

*Immunocytochemical detection of p53 protein in SCC and SCC cell lines*

Table I shows the results obtained when frozen sections of SCC from various head and neck sites were tested for overex-

**Table I** Immunocytochemical detection of p53 by monoclonal antibody p1801 in human squamous cell carcinomas

| *Tumour designation* | *Site* | *Stage (TNM)* | *Treatment prior to surgery* | *p1801 Reactivity* | *Total positive* |
|---|---|---|---|---|---|
| BICR-1 | Tongue | $T_2N_0M_0$ | None | + | |
| BICR-2 | Tongue | $T_1N_0M_0$ | None | − | |
| BICR-4 | Tongue | $T_4N_{2C}M_0$ | None | + | |
| BICR-5 M | Tongue | $T_4N_{2C}M_0$ | None | + | |
| BICR-7 | Tongue | $T_4N_{2B}M_0$ | None | + | |
| BICR-11 | Tongue | $T_3N_0M_0$ | None | − | 4/12 |
| BICR-16 R | Tongue | $T_2N_0M_0$ | Surgery and DXT | − | |
| BICR-20 | Tongue | $T_2N_0M_0$ | None | − | |
| BICR-21 | Tongue | $T_4N_3M_0$ | None | − | |
| BICR-22 M | Tongue | $T_4N_3M_0$ | None | − | |
| BICR-23 | Tongue | $T_4N_{2B}M_0$ | DXT | − | |
| BICR-24 | Tongue | $T_{1/2}N_0M_0$ | None | − | |
| BICR-19 | Epidermis | N/A | None | − | 0/1 |
| BICR-3 | Alveolus | $T_2N_{2B}M_0$ | None | + | |
| BICR-12 | Alveolus | $T_4N_3M_0$ | None | − | 1/2 |
| BICR-6 | Hypopharynx | $T_4N_1M_0$ | None | − | |
| BICR-15 | Larynx | $T_3N_0M_0$ | None | + | 1/4 |
| BICR-17 | Larynx | $T_4N_1M_0$ | None | − | |
| BICR-18 M | Larynx | $T_4N_1M_0$ | None | − | |
| BICR-9 | Retromolar | $T_3N_0M_0$ | None | + | 1/1 |
| BICR-10 R | Buccal Mucosa | $T_4N_0M_0$ | Surgery and DXT | − | 0/1 |
| BICR-8 | Floor of mouth | $T_4N_{2B}M_0$ | None | − | |
| BICR-13 R | Floor of mouth | $T_4N_{1B}M_0$ | Calcium implant | + | 1/3 |
| BICR-14 R | Floor of mouth | $T_3N_{2B}M_0$ | Radiotherapy | − | |

M = Lymph Node Metastasis    R = Recurrence    DXT = Deep X-ray Therapy pression of p53 protein. Altogether 8/24 tumours (33%) stained positive (four tongue, one larynx, one retromolar, one floor of mouth and one alveolus) and in the three cases where both the primary tumour and the corresponding lymph node metastases from the same patient were available for study the results obtained with the primary and metastatic lesions were the same (see also Figure 1). The staining was nuclear in all cases as reported previously (Gusterson *et al.*, 1991; Maestro *et al.*, 1992).

Eighteen SCC cell lines, including eight established from the above tumours (Table II, Figure 2), were also studied for the presence of elevated levels of p53 protein. Nine out of eighteen lines showed positive nuclear staining including 2/8 newly established cell lines from the above tumours (Table III). In these eight cases there was total agreement between the results obtained with a given cell line and the tumour from which is was derived (Table III). When protein samples obtained from the cell lines were analysed by Western blotting high steady state levels of p53 protein were observed only in the cell lines that showed a nuclear immunocytochemical staining pattern with p1801 (Figure 3, Table III).

*Direct sequencing of the p53 coding region in SCC cell lines and tumours*

As there are other possible mechanisms by which p53 protein could be stabilised other than by mutation we isolated mRNAs from several of the positively staining cell lines and subjected them to reverse transcription followed by PCR amplification and direct sequencing. All of these cell lines were found to contain point mutations or in-frame deletions within the coding region of the p53 gene (Table IV). Figure 4 shows the comparison of a mutant (CGC→CAC) p53 sequence found at codon 175 (arg→his) in line SCC-27 (Figure 4(a) as compared to the same region of wild type sequence found in line SCC-4 (Figure 4(b)). Of the thirteen mutations, eleven were apparently homozygous mutations since there was no evidence of expression of the normal p53 allele and seven of the point mutations have been described previously in other human tumours or cell lines (see Hollstein *et al.*, 1991; Caron de Fromental & Soussi, 1992 for reviews). We have also confirmed the mutation in line SCC-13 previously reported by Brash and co-workers (Brash *et al.*, 1991).

As with many p53 point mutations four of the seven (including SCC-13) resided within the conserved domains of the protein but one other occurred at codon 146 and two others at codon 151 in a region (amino acids 144–166) which has recently been suggested to be a 'hot spot' for tumours of non-small cell tumours of the lung (Mitsudomi *et al.*, 1992). Even more interestingly, all three of these mutations occurred in cell lines derived from SCCs of the tongue and all occurred at runs of Gs. The G→A transition at codon 151 (Proline to Histidine) has also been observed previously in an SCC of the epidermis (Brash *et al.*, 1991).

Five of the mutations were GC to AT transitions including one in line SCC-27 (from a metastatic vulval carcinoma) at a CpG site (Rideout *et al.*, 1990; Jones *et al.*, 1991). Three mutations were transversions; one GC→CG (BICR-3), one GC→TA (BICR-7) and one AT→CG (SCC-12 Clone B).

The eighth mutation, which occurred in a clone of the line SCC-12 originally derived from the facial epidermis of a kidney transplant patient (SCC-12 clone B; Rheinwald & Beckett, 1981; Parkinson *et al.*, 1983) is, to our knowledge, a novel T→G transversion at the second base of codon 216 which results in the substitution of valine with glycine. Of further interest is the observation that unusually the normal p53 allele was still expressed in SCC-12 Clone B. A non-tumorigenic sibling clone from the same tumour SCC-12 Clone F was shown to contain reproducibly less p53 protein as detected by Western blotting (Figure 3 compare lanes 4 and 8) and expression of the mutant p53 sequence was very difficult to detect in this clone although both alleles were present. This might reflect an imbalance in the expression of mutant and wild type p53 alleles in different clones of line

**Figure 1** Immunocytochemical detection of p53 protein in SCC frozen sections. Examples of cross-sections of invading portions of primary tongue SCCs BICR-1 **a**, BICR-4 **b**, and a lymph node metastasis BICR-4, BICR-5 **c**, stained using horseradish peroxidase catalysed visualisation of the monoclonal antibody p1801 (Banks *et al.*, 1986) using diamino benzidine. Bar = 35 μM; BV = Blood vessel; m = Mesenchyme; LN = Lymph node tissue. Note the absence of p53 staining in the more mature areas of tumour BICR4 (arrow).

SCC-12 and this is currently under investigation. Lines BICR-6 and BICR-16 possess stop codons at amino acids 192 and 146 and three other lines, BICR-22, SCC-25 and BICR-19 possess out of frame 19 bp, 2 bp and 107 bp deletions spanning codons 308+, 209+ and exon 10 respectively. A stop codon also resulted from the frame shift at codon 345 in line BICR22. Expression of a completely normal p53 allele in line BICR-19 was also detected. None of these five lines displayed elevated levels of p53 protein.

*Absence of detectable HPV16 and HPV18 DNA in SCC cell lines*

As it is possible that in the nine SCC lines we have studied where there is no elevation in p53 protein levels, p53 is being

**Table II** Squamous cell carcinoma cell lines used in this study

| Cell line designation | Site (TNM) | Stage | Treatment prior to surgery | Squamous origin | Tumorigenicity | Reference |
|---|---|---|---|---|---|---|
| SCC-4 | Tongue | Unknown | Radiation Methotrexate | Yes | Yes | Rheinwald & Beckett 1981 |
| SCC-9 | Tongue | | None | Yes | Yes | Rheinwald & Beckett 1981 |
| SCC-12 Clone B | Facial epidermis | N/A | None | Yes | Yes | Rheinwald & Beckett 1981 |
| SCC-13 | Epidermis | N/A | Radiation | Yes | Yes | Rheinwald & Beckett 1981 |
| SCC-15 | Tongue | Unknown | None | Yes | Yes | Rheinwald & Beckett 1981 |
| SCC-25 | Tongue | | None | Yes | Yes | Rheinwald & Beckett 1981 |
| SCC-27 M | Vulva (Peritoneal Metastasis) | N/A | Unknown | Yes | N.d | Parkinson et al., 1983 |
| BICR-3 | Upper Alveolus | $T_2N_{2B}M_0$ | None | Yes | No | Edington & Parkinson unpublished data |
| BICR-6 | Hypopharynx | $T_4N_1M_0$ | None | Yes | Yes | Edington & Parkinson unpublished data |
| BICR-7 | Tongue | $T_4N_2BM_0$ | None | Yes | N.d | Edington & Parkinson unpublished data |
| BICR-10 R | Buccal Mucosa | $T_4N_0M_0$ | DXT | Yes | Yes | Edington & Parkinson unpublished data |
| BICR-16 R | Tongue | $T_2N_0M_0$ | DXT | Yes | Yes | Edington & Parkinson Unpublished data |
| BICR-18 R | Larynx (Lymph Node Metastasis) | $T_4N_1M_0$ | None | Yes | Yes | Edington & Parkinson unpublished data |
| BICR-19 | Ear Epidermis | N/A | None | Yes | Yes | Edington & Parkinson unpublished data |
| BICR-22 M | Tongue (Lymph Node Metastasis) | $T_4N_3M_0$ | None | Yes | Yes | Edington & Parkinson unpublished data |
| BICR-31 | Tongue | $T_4N_{2B}M_0$ | None | N.d | Yes | Edington & Parkinson unpublished data |
| BICR-37 M | Tongue (Lymph Node Metastasis) | $T_4N_{2C}M_0$ | None | N.d | N.d | Edington & Parkinson unpublished data |
| BICR-56 | Tongue | $T_4N_1M_0$ | None | N.d | Yes | Edington & Parkinson unpublished data |

M = Metastasis R = Derived from recurrent tumour DXT = Deep X-ray Therapy.

**Table III** Immunocytochemical localisation of p53 protein using monoclonal antibody p1801

| Cell line tumour | Positive staining for p53 cell line | p53 protein detectable by Western blot: Tumour | Cell line |
|---|---|---|---|
| SCC-4 | + | N.d | + |
| SCC-9 | – | N.d | – |
| SCC-12 Clone B | + | N.d | + |
| SCC-13 | + | N.d | + |
| SCC-15 | – | N.d | – |
| SCC-25 | – | N.d | – |
| SCC-27 | + | N.d | + |
| BICR-3 | + | + | + |
| BICR-6 | – | – | – |
| BICR-7 | + | + | + |
| BICR-10 | – | – | – |
| BICR-16 | – | – | – |
| BICR-18 | – | – | – |
| BICR-19 | – | – | – |
| BICR-22 | – | – | – |
| BICR-31 | + | N.d | N.d |
| BICR-37 | + | N.d | N.d |
| BICR-56 | + | N.d | N.d |

**Figure 2** Immunocytochemical detection of mutant p53 proteins in SCC cell lines. Cell lines stained with antibody p1801 followed by horseradish peroxidase as detailed in Figure 1. The cells were photographed under phase contrast optics (a.c.e) and then again under bright field optics (no counterstain–**b,d,f**). Panels **a** and **b**, SCC-4; panels **c** and **d**, SCC-9; panels **e** and **f**, BICR-3. No staining was seen when the first antibody was omitted. Bar = 31 μM.

**Figure 3** Western blotting of SCC p53 proteins. Western blots of NP40-extracted proteins from normal human keratinocytes and 15 of the SCC cell lines. Human fibroblasts and JW/2 colon carcinoma cells were used as controls for normal levels of and no p53 protein respectively. HT29 colon carcinoma was included as a control line which was known to express high levels of p53 protein (Rodrigues *et al.*, 1990). Lanes 1, 20 and 22, HT29 (positive control); Lane 2, Human embryo fibroblasts (normal control); Lane 3, SCC-27; Lane 4, SCC-12 clone F; Lane 5, SCC-13; Lane 6, SCC-25; Lane 7, SCC-15; Lane 8, SCC-12 clone B; Lane 9, SCC-9; Lane 10, SCC-4; Lanes 11 and 19, JW/2 (negative control); Lane 12, normal human epidermal keratinocytes; Lane 13, BICR 22; Lane 14, BICR 19; Lane 15, BICR 16; Lane 16, BICR 10; Lane 17, BICR 6; Lane 18, BICR 3; Lane 21, BICR 7. The upper arrow of each panel shows the position of the 68 kd molecular weight marker and the lower arrow shows the position of the 43 kd marker. When the antibody was omitted no signal could be detected.

**Figure 4** Direct sequencing of PCR-amplified, reverse transcribed p53 mRNA: **a**, SCC-27 showing codon 175 G→A mutation. **b**, Wild type sequence in the same region (SCC 4). **c**, SCC-12 clone B showing expression of wild-type (T) and mutant (G) alleles at codon 216. Arrows marks positions of mutant bands.

**Table IV** p53 Mutations detected in squamous cell carcinomas and cell lines

| *Cell line* | *Codon* | *Mutation* | *Amino acid change* | *Base change* | *Expression of normal allele* | *Region sequenced (codons)* |
|---|---|---|---|---|---|---|
| SCC-4 | 151 | CCC→TCC | pro→ser | C→T | No | 105–393 |
| SSC-9[b] | 274–285 | 32 bp deletion | 285+ out of frame | – | No | Jung *et al.* (1992) |
| SCC-25 | 209 | 2 bp deletion | 209+ out of frame | – | No | 171–217 |
| SCC-12 Clone B | 216 | GTG→GGG | val→gly | T→G | Yes | 1–393 |
| SCC-13 | 258 | GAA→AAA | glu→lys | G→A | No | 105–393 |
| SCC-27 | 175 | CGC→CAC | arg→hist | G→A | No | 105–393 |
| BICR-3[a] | 282 | CGG→CCG | arg→pro | G→C | No | 105–383 |
| BICR-6 | 192 | CAG→TAG | glu→stop | C→T | No | 105–393 |
| BICR-7[a] | 151 | CCC→CAC | pro→his | C→A | No | 105–393 |
| BICR-10 | None detected | | | | | 1–331 |
| BICR-16[a] | 146 | TGG→TGA | trp→stop | G→A | No | 105–228 |
| BICR-18 | None detected | | | | | 2–96; 171–335; 342–393 |
| BICR-19 | exon 10 deleted | 107 bp deletion | 332+ out of frame | – | Yes | 1–393 |
| BICR-22[a] | exon 8/9 splice site | 19 bp deletion | 308+ out of frame→345 stop | – | No | 2–335 |
| BICR-31[a] | 173, 174 | 3 bp deletion | val arg→gly | TGA del. | No | 2–335 |
| BICR-56[a] | 126–132 | 21 bp deletion | 7aa deleted | – | No | 111–228 |

[a]Also detected in the original tumour sample [b]Data from Jung *et al.* (1992).

inactivated by degradation after complexing with an HPV virus such as HPV16 or HPV18 (Werness *et al.*, 1990; Scheffner *et al.*, 1990; Crook *et al.*, 1991*a*), we screened our cell lines for the presence of these viruses.

All of our cell lines were screened for the presence of HPV16 and 18 DNA by PCR, using DNA from the cell line SiHa as a positive control for a single copy of integrated HPV16 DNA. W12 and HeLa were used as further positive controls for HPV16 and 18 respectively. HPV-compatible, hypoxanthine guanosine phosphoribosyl transferase (HGPRT) primers were used to control for the integrity of each DNA sample and produced a band of approximately 275 bp in all of the SCC samples (Figure 5). Figure 5 also shows that in all of the SCC lines which stained negatively for p53 protein the 165 bp HPV16 and 99 bp HPV18 PCR products were absent. The faint band in the BICR16 track was not reproducible and was in any case too large to be the HPV16 product. This product may be indicative of a low copy number of another closely related HPV type and this is currently being investigated. Other HPV types which have been reported from the oral cavity such as HPV6, HPV-11 (Loning *et al.*, 1985) and HPV-4 (Yeudall & Campo, 1991) are also undetectable in lines SCC-4, SCC-9, SCC-12B, SCC-12F, SCC-13, SCC-15 and SCC-25 (M. Stanley-personal communication; A. Yeudall-personal communication). These data do not therefore support a role of these viruses in the inactivation of p53 in our series of SCC cell lines.

**Figure 5** HPV screening of BICR cell lines. Lane 1, φX 174 *Hae*III digested DNA molecular weight markers; lane 2, bacteriophage λ DNA positive control; lane 3, HeLa genomic DNA; lane 4, W12 genomic DNA; lanes 5–11, genomic DNA from BICR cell lines 3, 6, 10, 16, 18, 19 and 22 respectively. Band A represents the bacteriophage λ PCR product, B the HPRT gene PCR product, C the HPV 16 PCR product and D the HPV 18 PCR product.

### Discussion

We have confirmed the earlier reports of Field *et al.* (1991), Gusterson *et al.* (1991) and Maestro *et al.* (1992) that

• Biotinylated primer

▬ Position of highly conserved domains in protein

♦ Mutation sites

B2U Brash exon 2 primer

**Figure 6** Primers used for PCR of human p53 segments and the distribution of mutations found in SCC cell lines.

elevated levels of p53 protein are common in SCCs of the epidermis and oral cavity. Using a much larger series than that studied by Gusterson *et al.*, we also report a high frequency of p53 staining in cell lines established from SCCs including eight of the SCCs that were studied *in vivo*.

Our results are also in agreement with the statements made by those authors that there is no obvious correlation between the presence of high levels of p53 protein and tumour stage or treatment history and with the observation made by Gusterson *et al.* (1991) that the most intense staining was often observed in keratinocytes occupying a more basal position, or at the invading edge of the tumour (Figure 1, see also Purdie *et al.*, 1991). These results are in keeping with a role for p53 as a regulator of the cell cycle since in both normal and malignant epithelia, proliferation is restricted to the less differentiated cells.

Although in several human tumour systems a good correlation has been noted between the presence of elevated p53 protein levels and the presence of mutations within the coding region of the gene, this had not been investigated for SCC of the epidermis and oral cavity. This is important for the oral cavity in particular where increased p53 protein levels could result from complexing with the HPV E6 protein of HPV types 6 or 11 (Crook *et al.*, 1991*a*) or be secondary to another oncogenic event. Indeed, helix-loop-helix proteins have been suggested as potential regulators of p53 transcription (Ronen *et al.*, 1991) and one member of this protein family (c-*myc*) is frequently amplified in SCC of the head and neck (Yokota *et al.*, 1986). Furthermore, the *mdm*2 oncogene is known to bind and stabilise the p53 protein in tumours where p53 mutations are absent (Oliner *et al.*, 1992) and elevated p53 levels may also be a consequence of genetic instability (Lu *et al.*, 1992).

We have so far sequenced the p53 coding region of 14 SCC cell lines including eight of the nine lines which expressed high levels of p53 protein. In all cases where elevated levels of p53 protein were detected, mis-sense mutations, or in-frame deletions within the coding region were found, thus agreeing with the assertion that elevated levels of p53 protein are a good indicator of p53 mutation, at least in carcinomas of the head and neck region (see also Maestro *et al.*, 1992). In the case of the SCC of the upper aerodigestive tract, the mutations we detected were predominantly (4/5) G→A transitions or G→T transversions, consistent with the known action of benzo-(a)-pyrene and nitrosamines which are the most abundant classes of carcinogens found in cigarette smoke (IARC 1986, see also Maestro *et al.*, 1992). These observations are consistent with cigarette smoking being a major aetiological factor in the generation of these tumours (Stell, 1972). We also noted that many of the mutations or deletions detected in SCCs of the tongue (3/7) occurred within the region (codons 144–166) which has been reported to be a hot spot for non-small cell lung cancer (Mitsudomi *et al.*, 1992). Gusterson *et al.* (1991) also reported that one of two tongue SCC mutations occurred within this region, whereas (Maestro *et al.*, 1992) did not notice any in six of the larynx SCC mutations reported. Although the numbers are small, this might suggest that mutations within the region codons 144–166 are preferentially induced and/or selected at the tongue site, but this clearly needs further investigation. In 7/8 of the cases expression of the normal p53 allele was not detectable, thus indicating that loss of the normal p53 allele or an elimination of its expression had occurred during tumour progression. Normal allele expression was lost even in the case of line SCC-4 which has a proline→serine amino acid substitution at codon 151 of the p53 gene. This mutation has been reported to drive the wild type protein into the mutant conformation when the two are cotranslated *in vitro* (Milner & Medcalf, 1991). If this is the case in intact SCC-4 cells, some wild type p53 activity must remain in the heterozygous state, otherwise it is difficult to explain how the cells which had lost wild-type p53 expression gained a selective advantage in the tumour. In clone SCC-12 clone B however, both the mutated and normal p53 alleles were expressed. Cell line SCC-12 contains populations of tumorigenic (e.g. SCC-12 clone B) and non-tumorigenic (e.g. SCC-12 clone F) keratinocytes the former of which (but not the latter) possess a defect in their ability to respond to terminal differentiation stimuli (Rheinwald & Beckett, 1980; Parkinson *et al.*, 1983). Interestingly, we have found it difficult to detect the mutant p53 allele in the non-tumorigenic clone F of SCC-12 and this clone also contains far less p53 protein than SCC-12 clone B (Figure 3 compare lane 4 with lane 8). The heterozygous p53 mutation in line SCC-12 may therefore be an example of a p53 mutation which occurred late in SCC progression and its role in affecting keratinocyte behaviour is currently being explored. The mutation in SCC-12 clone B is to our knowledge novel and is of additional interest since the T→G transversion in the second base of codon 216 results in the substitution of a glycine for a valine and glycine substitutions have been proposed to introduce folds into amino acid chains thus altering protein conformation (see Gordon *et al.*, 1988). Several other types of amino acid substitution have previously been noted at codon 216 (Caron de Fromentel & Soussi, 1992), indicating that this part of the p53 molecule is a relevant site for mutagenesis.

We have also begun to investigate other possible mechanisms of p53 inactivation in our negatively-staining cell lines. Lines BICR-6 and BICR-16 were found to contain stop codon mutations and lines SCC-25, BICR-19 and BICR-22 out-of-frame deletions. Line SCC-9 (Table IV) was also recently reported to possess an out of frame deletion (Jung *et al.*, 1992). Since high levels of proteins were not observed in these lines it seems that these mutant proteins were not stabilised. This would be consistent with the absence of the sequences required for oligomerisation at the carboxy terminus (Vogelstein & Kinzler, 1992) as the epitope for antibody p1801 is close to the amino terminus of the p53 protein (Banks *et al.*, 1986) and should detect the truncated proteins if they were present. The other three negatively staining cell lines are currently being sequenced (see Table IV) to determine whether further stop codon mutations are present or whether mis-sense mutations that do not result in elevated levels of p53 protein are present (Halevy *et al.*, 1990; Malkin *et al.*, 1990). Both of these classes of mutations have already been reported in SCC of the epidermis (Brash *et al.*, 1991; Pierceall *et al.*, 1991) and head and neck (Sakai & Tsuchida, 1992). Also, mutations in the non-coding region of the gene may effect p53 transcription or translation and we are currently examining this possibility by Northern and Western blotting.

Finally, HPV types 16 and 18 are known to bind and degrade the p53 protein (Werness *et al.*, 1990; Scheffner 1990; Crook *et al.*, 1991*a*), so it is possible that any negatively-staining cell lines harbouring these HPV types could have inactivated p53 by this mechanism. This is particularly relevant to oral SCC since HPV16 and 18 are known to occur in the oral cavity (Maitland *et al.*, 1987; 1989; Yeudall & Campo, 1991) and can immortalise oral keratinocytes (Park *et al.*, 1991). Fifteen of the SCC lines under investigation (including all the negatively-staining ones) were repeatedly screened for HPV-16 and 18 DNA by PCR and were found to be negative. Therefore, p53 has not been inactivated by these HPV types in our cell lines. Our data do not rule out the possibility that there may be other HPV types capable to degrading p53 which are undetectable by the PCR primers we have used, but there is no evidence to support the existence of such viruses at present.

It has been suggested that *in vitro* immortalisation is connected with an important and possibly rate-limiting step in carcinogenesis (Newbold, 1985), although this has been disputed (Weinberg, 1989; Hunter, 1991). It is nevertheless interesting that a high frequency of p53 alterations are demonstrable both *in vivo* (Brash *et al.*, 1991; Pierceall *et al.*, 1991; Field *et al.*, 1991; Gusterson *et al.*, 1991, Sakai & Tsuchida, 1991; Maestro *et al.*, 1992; Brachman *et al.*, 1992, this study) and *in vitro* (Gusterson *et al.*, 1991; Sakai & Tsuchida, 1991, this study) in a cell type which frequently displays the property of *in vitro* immortalisation (Easty *et al.*, 1981; Rheinwald & Beckett, 1981). It is also noteworthy that fibroblasts derived from individuals with the Li-Fraumeni syndrome carrying germ-line p53 mutations are prone to spontaneous *in vitro* immortalisation (Bischoff *et al.*, 1990) and when mouse (Harvey & Levine, 1991) or chicken (Ulrich *et al.*, 1992) cells spontaneously immortalise *in vitro* p53 alterations are usually seen.

The current series of human SCC cell lines offer an opportunity to investigate the role of p53 in the pathogenesis of human SCC including its possible role in cellular immortalisation.

We wish to thank Professor Norman Maitland, York University for the gift of the HGPRT primers and advice concerning HPV screening, Dr Christos Paraskeva, Bristol University for lines JW-2 and HT29, and Ian McMillan of the Glasgow Veterinary School for preparing frozen sections of the human SCCs. These investigations were supported by grants awarded to the Beatson Institute by the Cancer Research Campaign.

### References

BAKER, S.J., FEARON, E.R., NIGRO, J.M., HAMILTON, S.R., PREISINGER, A.C., JESSUP, J.M., VAN TUINEN, P., LEDBETTER, D.H., BARKER, D.F., NAKAMURA, Y., WHITE, R. & VOGELSTEIN, B. (1989). Chromosome 17 deletions and p53 gene mutations in colorectal carcinomas. *Science*, **244**, 217–221.

BAKER, S.J., MARKOVITZ, S., FEARON, E.R., WILLSON, J.K. & VOGELSTEIN, B. (1990). Suppression of human colorectal carcinoma cell growth by wild-type p53. *Science*, **249**, 912–915.

BANKS, L., MATLASHEWSKI, G. & CRAWFORD, L. (1986). Isolation of human-p53-specific monoclonal antibodies and their use in the studies of human p53 expression. *Eur. J. Biochem.*, **259**, 529–534.

BARTEK, J., BARTKOVA, J., VOJTESEK, B., STADKOVA, Z., REJTHAR, A., KOVARIK, J. & LANE, D.P. (1990*a*). Patterns of expression of the p53 tumour suppressor in human breast tissues and tumours *in situ* and *in vitro*. *Int. J. Cancer*, **46**, 839–844.

BARTEK, J., IGGO, R., GANNON, J. & LANE, D.P. (1990*b*). Genetic and immunochemical analysis of mutant p53 in human breast cancer cell lines. *Oncogene*, **5**, 893–899.

BENNETT, W.P., HOLLSTEIN, M.C. HE, A., ZHU, S.M. RESAU, J.H. TRUMP, B.F., METCALF, R.A., WELSH, J.A., MIDGLEY, C., LANE, D.P. & HARRIS, C.C. (1991). Archival analysis of p53 genetic and protein alterations in Chinese esophageal cancer. *Oncogene*, **6**, 1779–1784.

BISCHOFF, F.Z., STRONG, L.C., YIM, S.O., PRATT, D.R., SICILIANO, M.J., GIOVANELLA, B.C. & TAINSKY, M.A. (1991). Tumorigenic transformation of spontaneously immortalized fibroblasts from patients with a familial cancer syndrome. *Oncogene*, **6**, 183–186.

BISCHOFF, F.Z., YIM, S.O., PATHAK, S., GRANT, G., SICILIANO, M.J., GIOVANELLA, B.C., STRONG, L.C. & TAINSKY, M.A. (1990). Spontaneous abnormalities in normal fibroblasts from patients with Li-Fraumeni cancer syndrome: aneuploidy and immortalization. *Cancer Res.*, **50**, 7979–7984.

BRACHMAN, D.G., GRAVES, D., VOKES, E., BECKETT, M., HARAF, D., MONTAG, A., DUNPHY, E., MICK, R., YANDELL, D. & WEICHSELBAUM, R.R. (1992). Occurance of p53 gene deletions and human papilloma virus infection in human head and neck cancer. *Cancer Res.*, **52**, 4832–4836.

BRASH, D.E., RUDOLPH, J.A., SIMON, J.A., LIN, A., McKENNA, G.J., BADEN, H.P., HALPERIN, A.J. & PONTEN, J. (1991). A role for sunlight in skin cancer: UV-induced p53 mutations in squamous cell carcinomas. *Proc. Natl Acad. Sci. USA*, **88**, 10124–10128.

CARON DE FROMENTEL, C. & SOUSSI, T. (1992). TP53 Tumor suppressor gene – a model for investigating human mutagenesis. *Genes Chromosomes & Cancer*, **4**, 1–15.

CASEY, G., LU-HSUEH, M., LOPEZ, M.E., VOGELSTEIN, B. & STANBRIDGE, E.J. (1991). Growth suppression of human breast cancer cells by the introduction of a wild-type p53 gene. *Oncogene*, **6**, 1791–1797.

CATTORETTI, G., RILKE, F., ANDRELOA, S., D'AMATO, L. & DELIA, D.A (1988). P53 expression in breast cancer. *Int. J. Cancer*, **41**, 178–183.

CHEN, P.L., CHEN, Y., BOOKSTEIN, R. & LEE, W.H. (1990). Genetic mechanisms of tumor suppression by the human p53 gene. *Science*, **250**, 1576–1580.

CHEN, Y., CHEN, P.L., ARNAIZ, N., GOODRICH, D. & LEE, W.H. (1991). Expression of wild-type p53 in human A673 cells suppresses tumorigenicity but not growth rate. *Oncogene*, **6**, 1799–1805.

CHENG, J., YEE, J.K., YEARGIN, J., FRIEDMANN, T. & HASS, M. (1992). Suppression of acute lymphoblastic leukemia by the human wild-type p53 gene. *Cancer Res.*, **52**, 222–226.

CHIBA, I., TAKAHASHI, T., NAU, M.M., D'AMICO, D., CURIEL, D.T., MITSUDOMI, T., BUCHHAGEN, D.L. CARBONE, D., PIANTODOSI, S., KOGA, H., REISSMAN, P.T., SLAMON, D.J., HOLMES, E.C. & MINNA, J.D. (1990). Mutations in the p53 gene are frequent in primary, resected non-small cell lung cancer. *Oncogene*, **5**, 1603–1610.

CROOK, T., TIDY, J.A. & VOUSDEN, K.H. (1991*a*). Degradation of p53 can be targeted by HPV E6 sequences distinct from those required for p53 binding and transactivation. *Cell*, **67**, 547–556.

CROOK, T., WREDE, D., TIDY, J., SCHOLEFIELD, J., CRAWFORD, L. & VOUSDEN, K.H. (1991*b*). Status of c-myc, p53 and retinoblastoma genes in human papillomavirus positive and negative squamous cell carcinomas of the anus. *Oncogene*, **6**, 1251–1257.

DILLER, L., KASSEL, J., NELSON, C.E., GRYKA, M.A., LITWAK, G., GEBHARDT, M., BRESSAC, B., OZTARK, M., BAKER, S.J., VOGELSTEIN, B. & FRIEND, S.H. (1990). p53 functions as a cell cycle control protein in osteosarcomas. *Mol. Cell Biol.*, **10**, 5772–5781.

DONEHOWER, L.A., HARVEY, M., SLAGLE, B.L., MCARTHUR, M.J., MONTGOMERY, C.A. Jr., BUTEL, J.S. & BRADLEY, A. (1992). Mice deficient for p53 are developmentally normal but susceptible to spontaneous tumours. *Nature*, **356**, 215–221.

EASTY, D.M., EASTY, G.C., CARTER, R.L., MONOGHAN, P. & BUTLER, L.J. (1981). Ten human carcinoma cell lines derived from squamous carcinomas of the head and neck. *Br. J. Cancer*, **43**, 772–785.

FEARON, E.R., HAMILTON, S.R. & VOGELSTEIN, B. (1987). Clonal analysis of human colorectal tumours. *Science*, **238**, 193–197.

FIELD, J.K., SPANDIDOS, D.A., MALLIRI, A., GOSNEY, J.R., YIAGNISIS, M. & STELL, P.M. (1991). Elevated P53 expression correlates with a history of heavy smoking in squamous cell carcinoma of the head and neck. *Br. J. Cancer*, **64**, 573–577.

FINLAY, C.A., HINDS, P.W. & LEVINE, A.J. (1989). The p53 protooncogene can act as a suppressor of transformation. *Cell*, **57**, 1083–1093.

FINLAY, C.A., HINDS, P.W., TAN, T.H., ELIYAHU, D., OREN, M. & LEVINE, A.J. (1988). Activating mutations for transformation by p53 produce a gene product that forms an hsc70-p53 complex with an altered half-life. *Mol. Cell. Biol.*, **8**, 531–539.

GORDON, A.J.E., BURNS, P.A., FIX, D.F., YATAGAI, F., ALLEN, F.L., HORSFALL, M.J., HALLIDAY, J.A., GRAY, J., BERNELOT-MOENS, C. & GLICKMAN, B.W. (1988). Missense mutation in the lacI gene of Escherichia coli. Inferences on the structure of the repressor protein. *J. Mol. Biol.*, **200**, 239–251.

GREEN, H. (1978). Cyclic AMP in relation to proliferation of the epidermal cell: a new view. *Cell*, **15**, 801–811.

GUSTERSON, B.A., ANBAZHAGAN, R., WARREN, W., MIDGELY, C., LANE, D.P., O'HARE, M., STAMPS, A., CARTER, R. & JAYATILAKE, H. (1991). Expression of p53 in premalignant and malignant squamous epithelium. *Oncogene*, **6**, 1785–1789.

HALEVY, O., MICHAELOVITZ, D. & OREN, M. (1990). Different tumor-derived p53 mutants exhibit distinct biological activities. *Science*, **250**, 113–116.

HARVEY, D.M. & LEVINE, A.J. (1991). p53 alteration is a common event in the spontaneous immortalisation of primary BALB. C murine embryo fibroblasts. *Genes & Development*, **5**, 2375–2385.

HAWLEY-NELSON, P., VOUSDEN, K.H., HUBBERT, N.L., LOWY, D.R. & SCHILLER, J.T. (1989). HPV16 E6 and E7 proteins cooperate to immortalize human foreskin keratinocytes. *EMBO J.*, **8.**, 3905–3910.

HOLLSTEIN, M., METCALF, R.A., WELSH, J.A., MONTESANO, R. & HARRIS, C.C. (1990). Frequent mutation of the p53 gene in human esophegeal cancer. *Proc. Natl Acad. Sci. USA*, **87**, 9958–9961.

HOLLSTEIN, M., SIDRANSKY, D., VOGELSTEIN B. & HARRIS, C.C. (1991). p53 mutations in human cancers. *Science*, **253**, 49–53.

HUNTER, T. (1991). Cooperation between oncogenes. *Cell*, **64**, 249–270.

HURLIN, P.J., KAUR, P., SMITH, P.P., PEREZ-REYES, N., BLANTON, R.A. & McDOUGALL, J.K. (1991). Progression of human papillomavirus type 18-immortalized human keratinocytes to a malignant phenotype. *Proc. Natl Acad. Sci. USA*, **88**, 570–574.

IARC (1986). Monographs on the evaluation of the carcinogenic risk of chemicals to humans. Vol. **38**, *Tobacco Smoking* IARC Lyon.

IGGO, R., GATTER, K., BARTEK, J., LANE, D. & HARRIS, A.L. (1990). Increased expression of mutant forms of p53 oncogene in primary lung cancer. *Lancet*, **335**, 675–679.

JONES, P.A., BUCKLEY, J.D., HENDERSON, B.E., ROSS, R.K. & PIKE, M.C. (1991). From gene to carcinogen: a rapidly evolving field in molecular epidemiology. *Cancer Res.*, **51**, 3617–3620.

JUNG, M., NOTARIO, V. & DRITSCHILO, A. (1992). Mutations in the p53 gene in radiation-sensitive and -resistant human squamous carcinoma cells. *Cancer Res.*, **52**, 6390–6393.

KAUR, P. & McDOUGALL, J.K. (1988). Characterization of primary keratinocytes transformed by human papillomavirus type 18. *J. Virol.*, **62**, 1917–1924.

LANE, D.P. & CRAWFORD, L.V. (1979). T antigen is bound to a host protein in SV40 transformed cells. *Nature*, **278**, 261–263.

LAW, J.C., STRONG, L.C., CHIDAMBARAM, A. & FERRELI, R.E. (1991). A germ line mutation in exon 5 of the p53 gene in an extended cancer family. *Cancer Res.*, **51**, 6385–6387.

LI, F.P. & FRAUMENI, J.F. Jr. (1969). Rhabdomyosarcoma in children: epidemiologic study and identification of a familial cancer syndrome. *J. Natl Cancer Inst.*, **43**, 1365–1373.

LINZER, D.I. & LEVINE, A.J. (1979). Characterization of a 54K Dalton cellular SV40 tumor antigen present in SV40-transformed cells and uninfected embryonal carcinoma cells. *Cell*, **17**, 43–52.

LONING, T., IKENBERG, H., BECKER, J., GISSMAN, L., HOEPFNER, I. & ZUR HAUSEN, H. (1985). Analysis of oral papillomas, leukoplakias and invasive carcinomas for human papillomavirus type related DNA. *J. Invest. Dermatol.*, **88**, 417–420.

LU, X., PARK, S.H., THOMPSON, T. & LANE, D.P. (1992). ras-induced hyperplasia occurs with mutation of p53 but activated ras and myc together can induce carcinoma without p53 mutation. *Cell*, **70**, 153–161.

MAESTRO, R., DOLCETTI, R., GASPAROTTO, C., DOGLIONI, C., PELUCCHI, S., BARZAN, L., GRANDI, E. & BOIOCCHI, M. (1992). High frequency of p53 gene alterations associated with protein overexpression in human squamous cell carcinoma of the larynx. *Oncogene*, **7**, 1159–1166.

MAITLAND, N.J., COX, M.F., LYNAS, C., PRIME, S.S. & SCULLY, C. (1987). Detection of human papillomavirus DNA in biopsies of human oral tissue. *Br. J. Cancer*, **56**, 245–250.

MAITLAND, N.J., BROMIDGE, T., COX, M.F., CRANE, I.J., PRIME, S.S. & SCULLY, C. (1989). Detection of human papillomavirus genes in human oral tissue biopsies and cultures by polymerase chain reaction. *Br. J. Cancer*, **59**, 698–703.

MALKIN, D., LI, F.P., STRONG, L.C., FRAUMENI, J.F. Jr., NELSON, C., KIM, D.M., KASSEL, J., GRYKA, M.A., BISCHOFF, F.Z., TAINSKY, M.A. & FRIEND, S.H. (1990). Germ line p53 mutations in a familial syndrome of breast cancer, sarcomas and other neoplasms. *Science*, **250**, 1233–1238.

MERCER, W.E., SHIELDS, M.T., AMIN, M., SAUVE, G.J., APPELLA, E., ROMANO, J.W. & ULLRICH, S.J. (1990). Negative growth regulation in a glioblastoma tumor cell line that conditionally expresses human wild type p53. *Proc. Natl Acad. Sci. USA*, **87**, 6166–6170.

MICHAELOVITZ, D., HALEVY, O. & OREN, M. (1990). Conditional inhibition of transformation and of cell proliferation by a temperature-sensitive mutant of p53. *Cell*, **62**, 671–680.

MILNER, J. & MEDCALF, E.A. (1991). Cotranslation of activated mutant p53 with wild type drives the wild-type p53 protein into the mutant conformation. *Cell*, **65**, 765–774.

MITSUDOMI, T., STEINBERG, S.M., NAU, M.M., CARBONE, D., D'AMICO, D., BODNER, S., OIE, H.K., LINNIOLA, I., MULSHINE, J.L., MINNA, J.D. & GAZDAR, A.F. (1992). p53 gene mutations in non-small-cell lung cancer cell lines and their correlation with the presence of *ras* mutations and clinical features. *Oncogene*, **7**, 171–180.

MUNGER, K., PHELPS, W.C., BUBB, V., HOWLEY, P.M. & SCHEGEL, R. (1989). The E6 and E7 genes of the human papillomavirus type 16 together are necessary and sufficient for transformation of primary human keratinocytes. *J. Virol.*, **63**, 4417–4421.

NEWBOLD, R.F. (1985). Multistep malignant transformation of mammalian cells by carcinogens: induction of immortality as a key event. In Carcinogenesis: a Comprehensive Survey Vol. 9 *Mammalian Cell Transformation: Mechanisms of Carcinogenesis and Assays for Carcinogens* pp. 17–28. Barratt, J.C. & Tennant, R.W. (eds.) Raven Press: New York.

NIGRO, J.M., BAKER, S.J., PREISINGER, A.C., JESSUP, J.M., HOSTETTER, R., CLEARY, K., BIGNER, S.H., DAVIDSON, N., BAYLIN, S., DEVILEE, P., GLOVER, T., COLLINS, F.S., WESTON, A., MODALI, R., HARRIS, C.C. & VOGELSTEIN, B. (1989). Mutations in the p53 gene occur in diverse human tumour types. *Nature*, **342**, 705–708.

OLINER, J.D., KINZLER, K.W., MELTZER, P.S., GEORGE, D.L. & VOGELSTEIN, B. (1992). Amplification of a gene encoding a p53-associated protein in human sarcomas. *Nature*, **358**, 80–83.

OREN, M., MALTZMAN, W. & LEVINE, A.J. (1981). Post-translational regulation of the 54K cellular antigen in normal and transformed cells. *Mol. Cell Biol.*, **1**, 101–110.

PARK, N.H., MIN, B.M., LI, S.L., HUANG, M.Z., CHERICK, H.M. & DONIGER, J. (1991). Immortalization of normal human oral keratinocytes with type 16 human papillomavirus. *Carcinogenesis*, **12**, 1627–1631.

PARKINSON, E.K., GRABHAM, P. & EMMERSON, A. (1983). A subpopulation of cultured human keratinocytes which is resistant to the induction of terminal differentiation-related changes by phorbol, 12-myristate, 13-acetate: evidence for an increase in the resistant population following transformation. *Carcinogenesis*, **4**, 857–861.

PARKINSON, E.K., HUME, W.J. & POTTEN, C.S. (1986). The radiosensitivity of cultured human and mouse keratinocytes. *Int. J. Rad. Biol.*, **50**, 717–726.

PATER, M.M. & PATER, A. (1985). Human papillomavirus types 16 and 18 sequences in carcinoma cell lines of the cervix. *Virology*, **145**, 313–318.

PIERCEALL, W.E., MUKHOPADHYAY, T., GOLDBERG, L.H. & ANANTHASWAMY, H.N. (1991). Mutations in the p53 tumor suppressor gene in human cutaneous squamous cell carcinomas. *Mol. Carcinog.*, **4**, 445–449.

PIRISI, L., YASUMOTO, S., FELLER, M., DONIGER, J. & DIPAULO, J.A. (1987). Transformation of human fibroblasts and keratinocytes with human papillomasvirus type 16 DNA. *J. Virol.*, **61**, 1061–1066.

PURDIE, C.A., O'GRADY, J., PIRIS, J., WYLLIE, A.H. & BIRD, C.C. (1991). p53 expression in colorectal tumors. *Am. J. Pathol.*, **138**, 807–813.

RHEINWALD, J.G. & BECKETT, M.A. (1980). Defective terminal differentiation in culture as a consistent and selectable character of malignant human keratinocytes. *Cell*, **22**, 629–632.

RHEINWALD, J.G. & BECKETT, M.A. (1981). Tumourigenic keratinocyte lines requiring anchorage and fibroblast support cultured from human squamous cell carcinomas. *Cancer Res.*, **41**, 1657–1663.

RHEINWALD, J.G. & GREEN, H. (1975). Serial cultivation of strains of human epidermal keratinocytes: the formation of keratinizing colonies from single cells. *Cell*, **6**, 331–343.

RIDEOUT, W.M.III, COETZEE, G.A., OLUMI, A.F. & JONES, P.A. (1990). 5-Methylcytosine as an endogenous mutagen in the human LDL receptor and p53 genes. *Science*, **249**, 1288–1290.

RODRIGUES, N.R., ROWAN, A., SMITH, M.E.F., KERR, I.B., BODMER, W.F., GANNON, J.V. & LANE, D.P. (1990). p53 mutations in colorectal cancer. *Proc. Natl Acad. Sci. USA*, **87**, 7555–7559.

RONEN, D., ROTTER, V. & REISMAN, D. (1991). Expression from the murine p53 promoter is mediated by factor binding to a downstream helix-loop-helix recognition motif. *Proc. Natl Acad. Sci. USA*, **88**, 4128–4132.

SAKAI, E. & TSUCHIDA, N. (1992). Most human squamous cell carcinomas in the oral cavity contain mutated p53 tumor-suppressor genes. *Oncogene*, **7**, 927–933.

SARNOW, P., HO, Y.S., WILLIAMS, J. & LEVINE, A.J. (1982). Adenovirus Elb-58 kd tumor antigen and SV40 large tumor antigen are physically associated with the same 54 kd cellular protein in mammalian cells. *Cell*, **28**, 387–394.

SCHEFFNER, M., MUNGER, K., BYRNE, J.C. & HOWLEY, P.M. (1991). The state of the p53 and retinoblastoma genes in human cervical carcinoma cell lines. *Proc. Natl Acad. Sci, USA*, **88**, 5523–5527.

SCHEFFNER, M., WERNESS, B.A., HUMBREGTSE, J.M., LEVINE, A.J. & HOWLEY, P.M. (1990). The E6 oncoprotein encoded by human papillomavirus types 16 and 18 promotes the degradation of p53. *Cell*, **63**, 1129–1136.

SRIVASTAVA, S., ZOU, Z.Q., PIROLLO, K., BLATTNER, W.A. & CHANG, E.H. (1990). Germ-line transmission of a mutated p53 gene in a cancer-prone family with Li-Fraumeni syndrome. *Nature*, **348**, 747–749.

STELL, P.M. (1972). Smoking and laryngeal cancer. *Lancet*, **i**, 617–618.

UICC (1987) Union Internationale Contre le Cancer *TNM Classification of Malignant Tumours* Hermanele, P. & Fobin, L. (eds.) Springer Verlag, Berlin.

ULRICH, E., BOEHMELT, G., BIRD, A. & BEUG, H. (1992). Immortalization of conditionally transformed chicken cells: loss of normal p53 expression is an early step that is independent of cell transformation. *Genes & Develop.*, **6**, 876–887.

VAN DER BERG, F.M., TIGGES, A.J., SCHIPPER, M.E., DEN HARTOG-JAGER, F.C.A., KROES, W.G.M. & WALBOOMERS, J.M.M. (1989). Expression of the nuclear oncogene p53 in colon tumours. *J. Pathol.*, **157**, 193–199.

VOGELSTEIN, B., FEARON, E.R., HAMILTON, S.R., KERN, S.E., PREISINGER, A.C., LEPPART, M., NAKAMURA, Y., WHITE, R., SMITS, A.M. & BOS, J.L. (1988). Genetic alterations during colorectal-tumor development. *New Eng. J. Med.*, **319**, 525–532.

VOGELSTEIN, B. & KINZLER, K.W. (1992). p53 function and dysfunction. *Cell*, **70**, 523–526.

WEINBERG, R.A. (1989). Oncogene, antioncogenes, and the molecular bases of multistep carcinogenesis. *Cancer Res.*, **49**, 3713–3721.

WERNESS, B.A., LEVINE, A.J. & HOWLEY, P.M. (1990). Association of human papillomavirus types 16 and 18 E6 proteins with p53. *Science*, **248**, 76–79.

WESTON, A., WILLEY, J.C., MODALI, R., SUGIMURA, H., MCDOWELL, E.M., RESAN, J., LIGHT, B., HAUGEN, A., MANN, D.L., TRUMP, B.F. & HARRIS, C.C. (1989). Differential DNA sequence deletions from chromosomes 3, 11, 13, and 17 in squamous-cell carcinoma, large-cell carcinoma, and adenocarcinoma of the human lung. *Proc. Natl Acad. Sci. USA*, **86**, 5099–5103.

WOLF, D., HARRIS, N. & ROTTER, V. (1984). Reconstitution of p53 expression in a non producer Ab-MuLV-transformed cell line by transfection of a functional p53 gene. *Cell*, **38**, 119–126.

YEUDALL, W.A. & CAMPO, M.S. (1991). Human papillomavirus DNA in biopsies of oral tissues. *J. Gen. Virol.*, **72**, 173–176.

YOKOTA, J., TSUNETSUGU-YOKOTA, Y., BATTIFORA, H., LE FEVRE, C. & CLINE, M.J. (1986). Alterations of myc, myb, and rasHa proto-oncogenes in cancers are frequent and show clinical correlation. *Science*, **231**, 261–265.

YOKOTA, J., WADA, M., SHIMOSATO, Y., TERADA, M. & SUGIMURA, T. (1987). Loss of heterozygosity of chromosomes 3, 13, and 17 in small-cell carcinoma and on chromosome 3 in adenocarcinoma of the lung. *Proc. Natl Acad. Sci. USA*, **84**, 9252–9256.


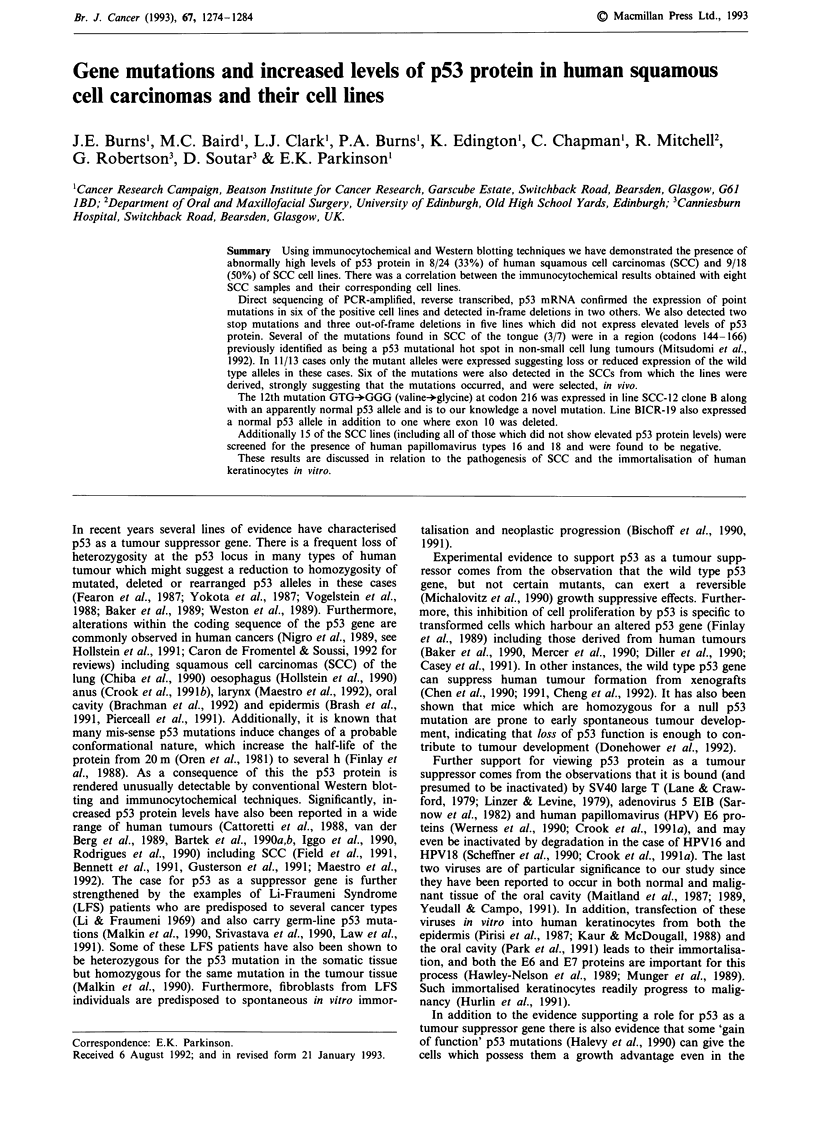

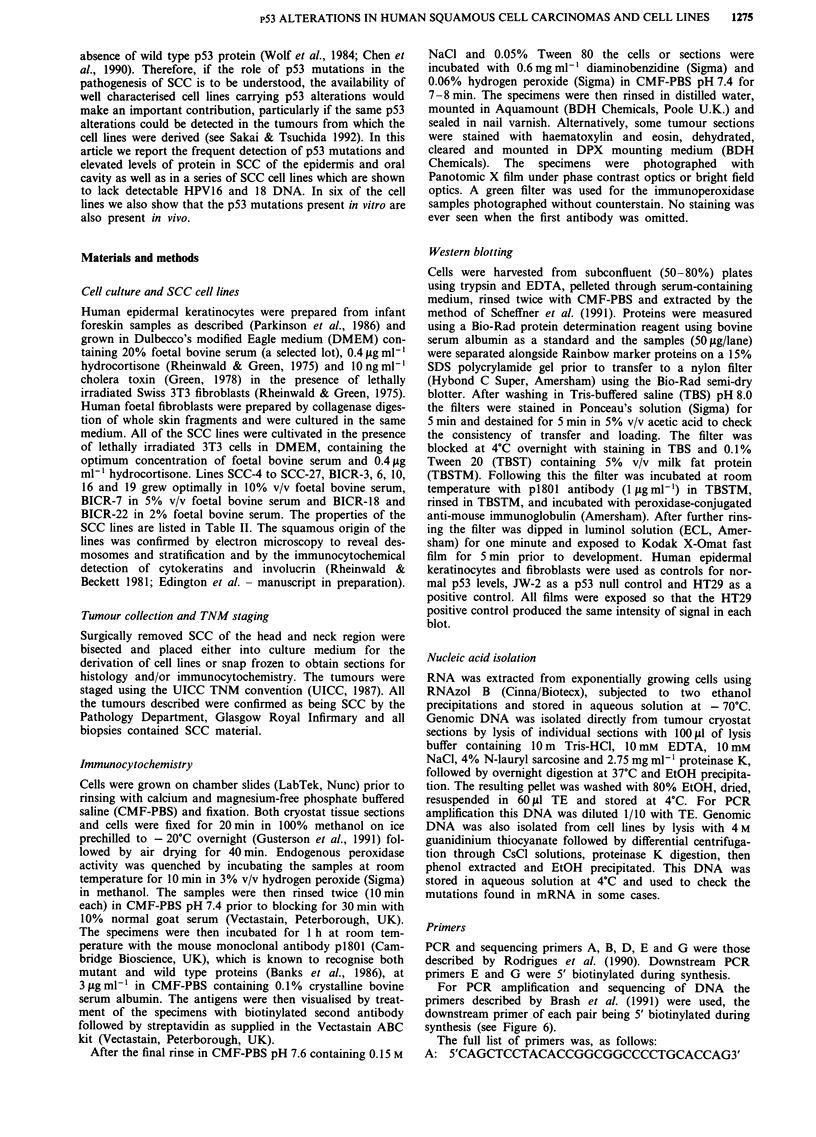

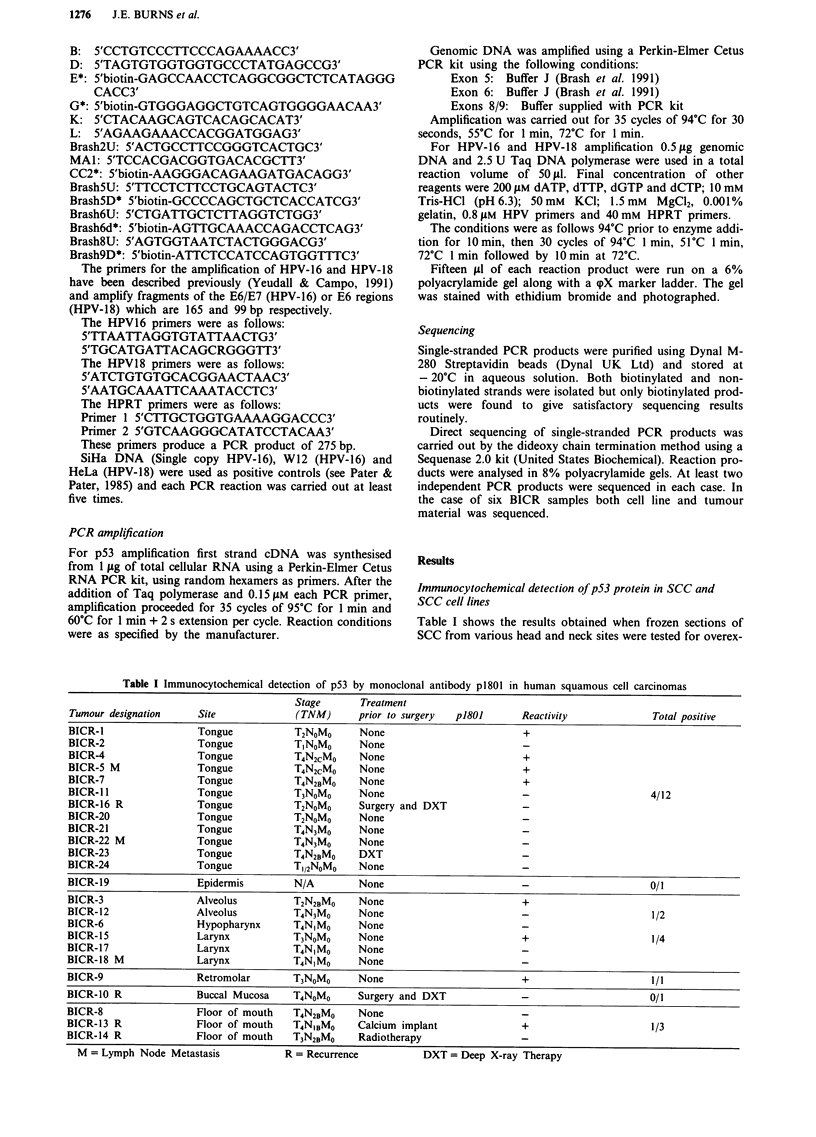

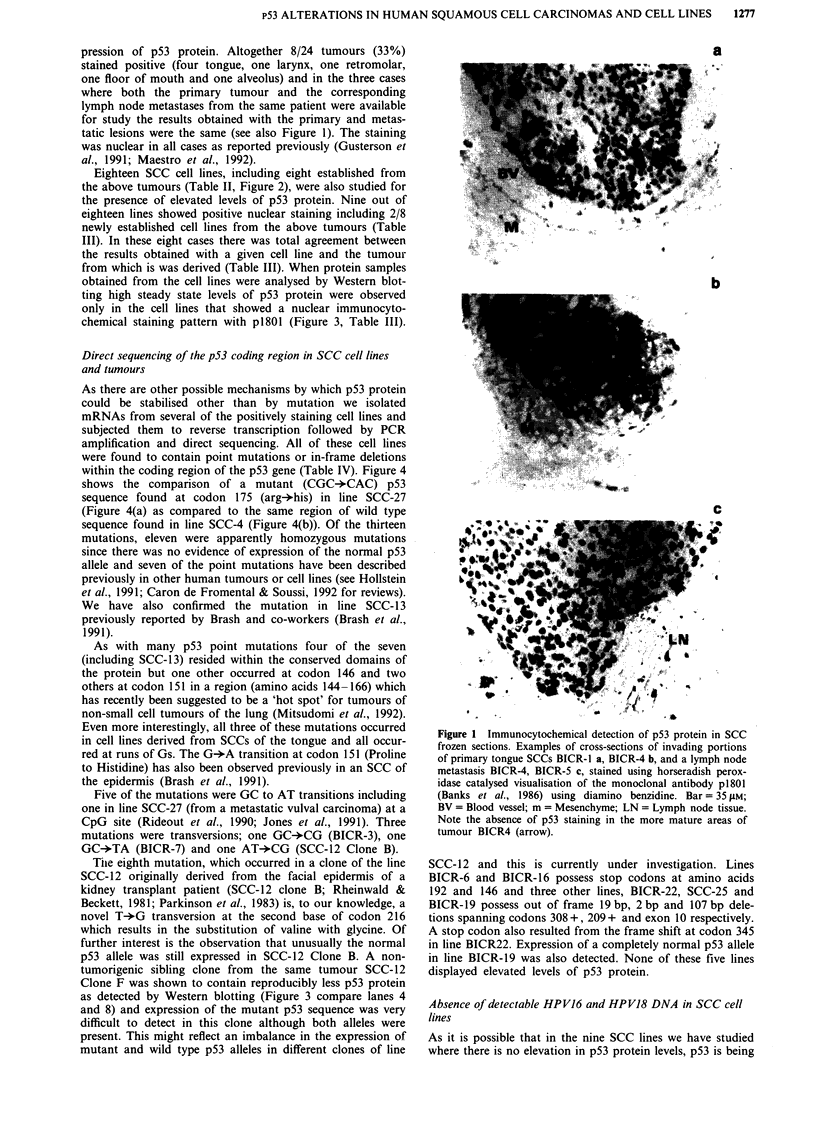

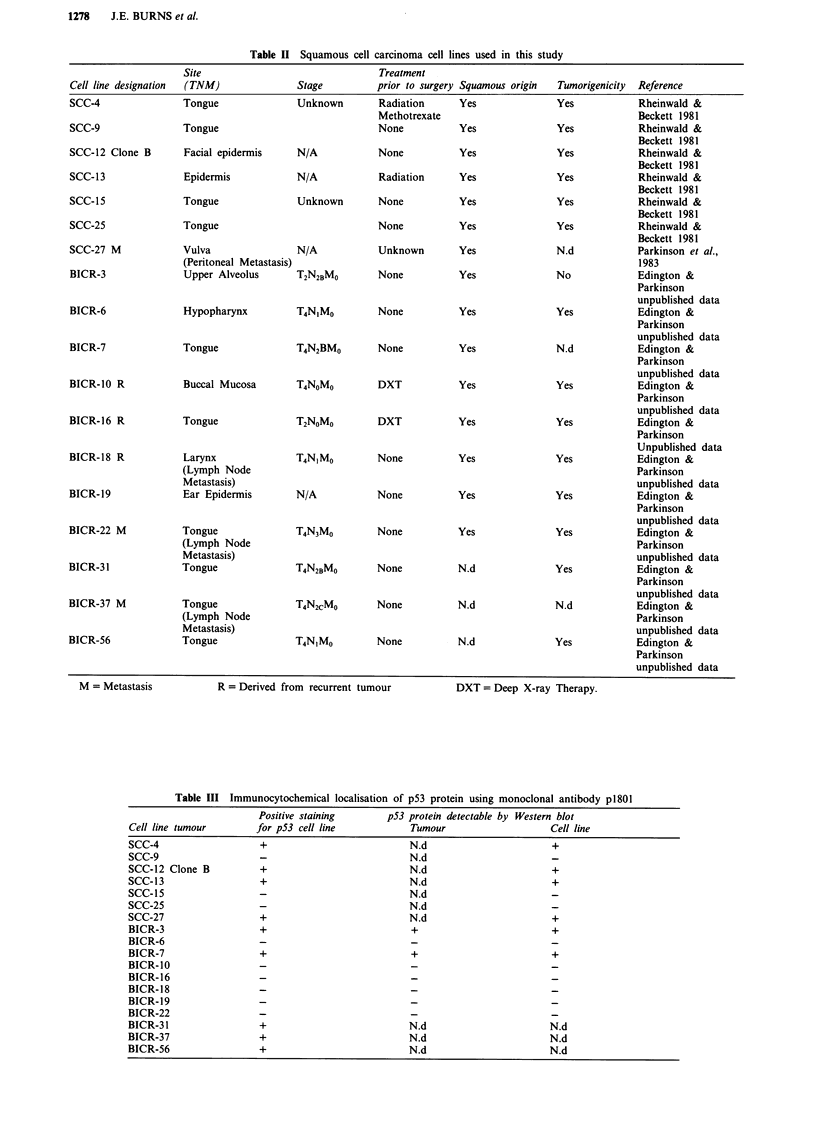

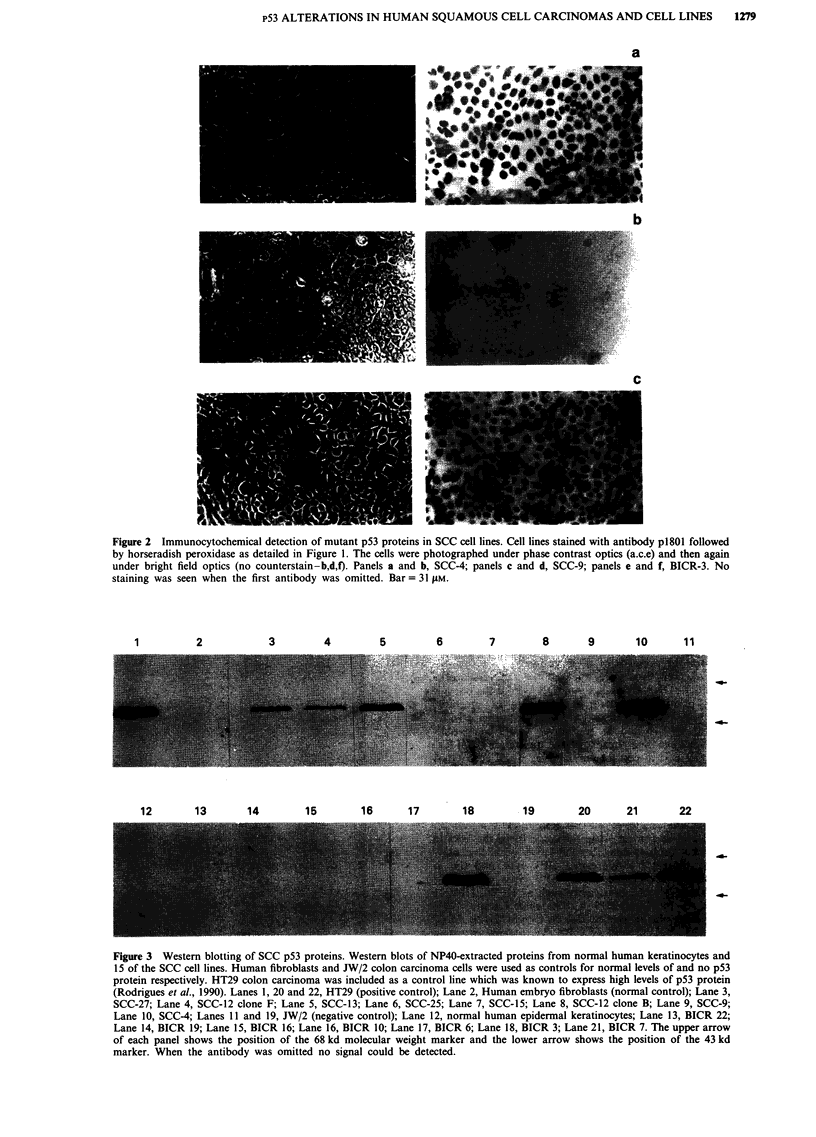

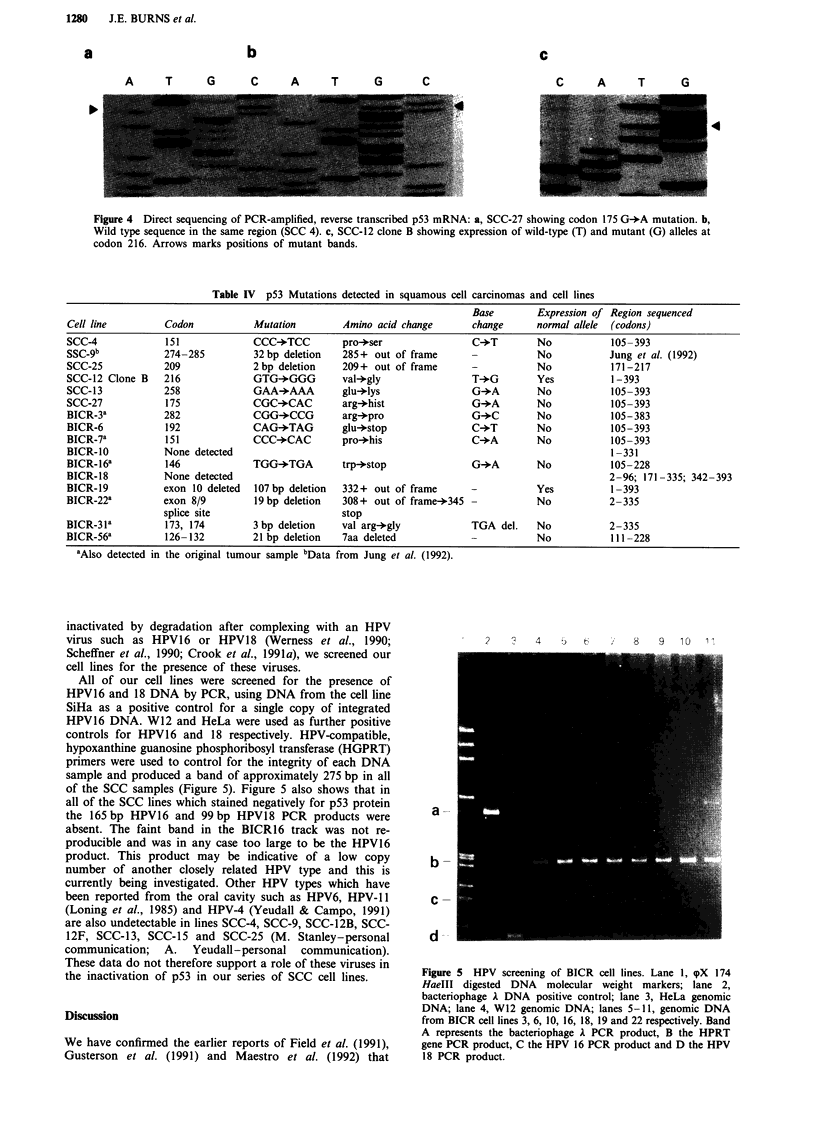

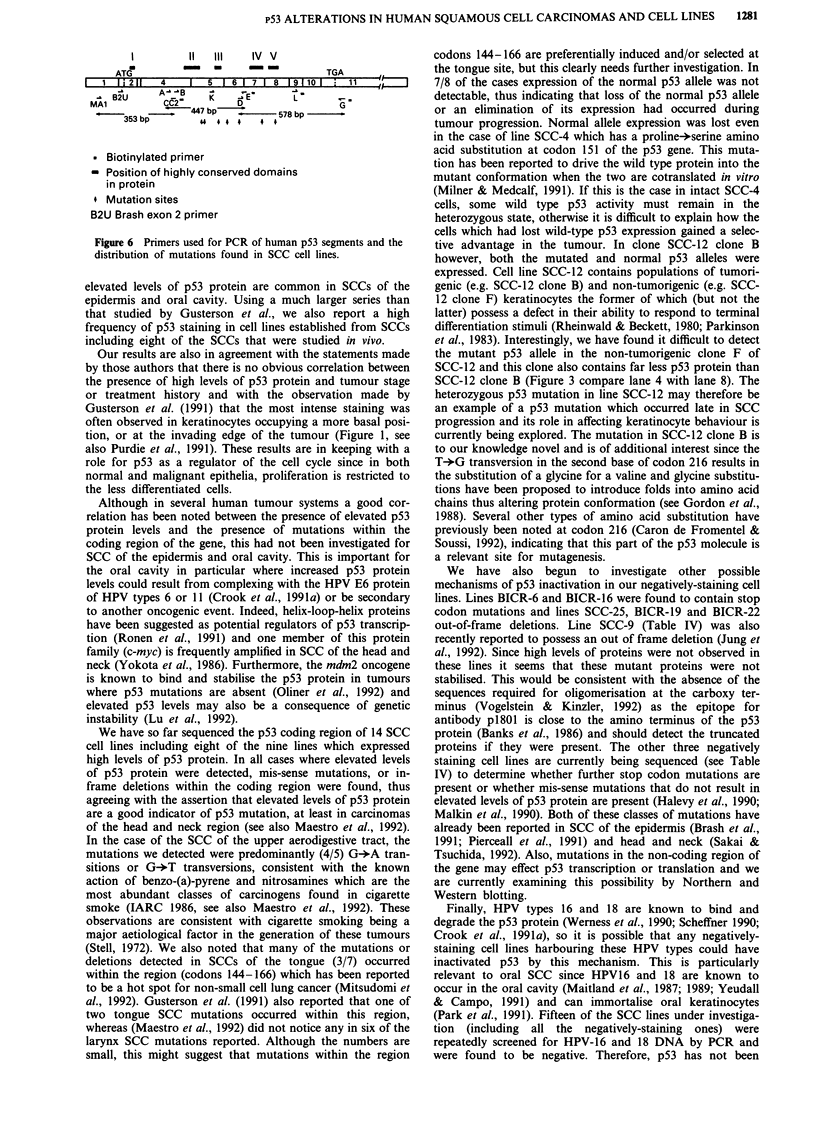

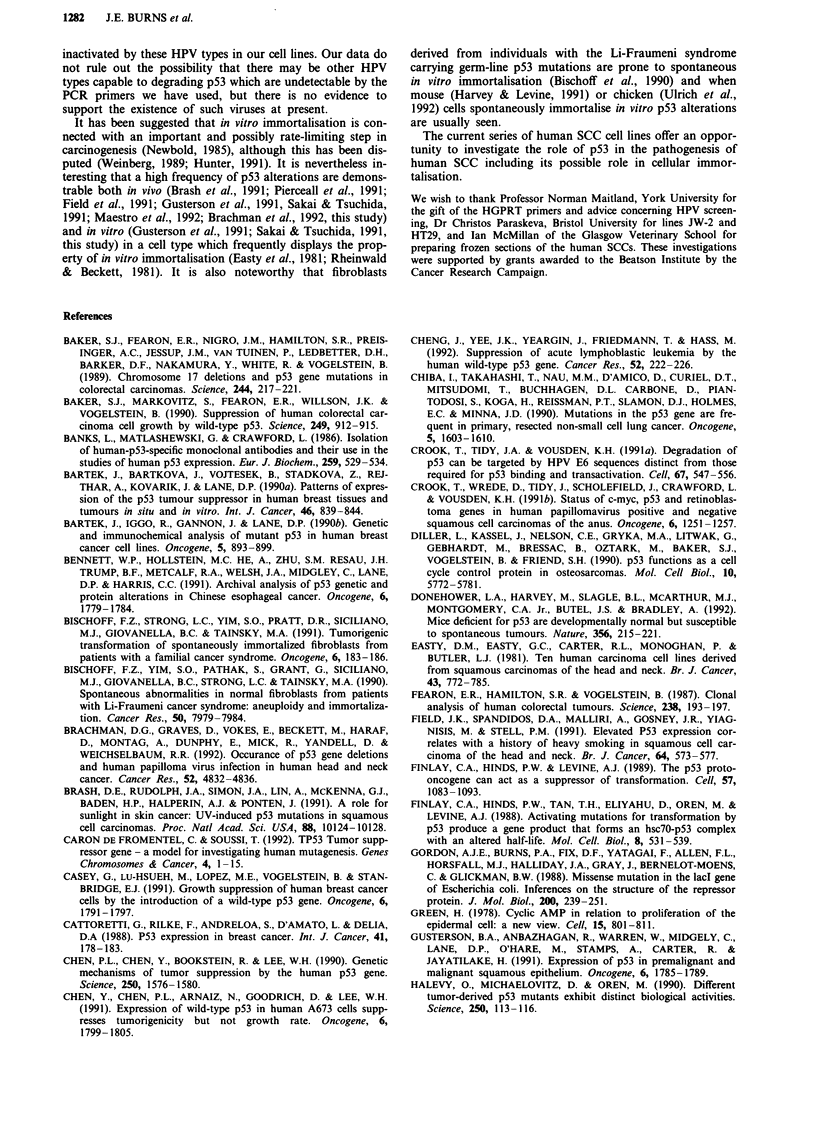

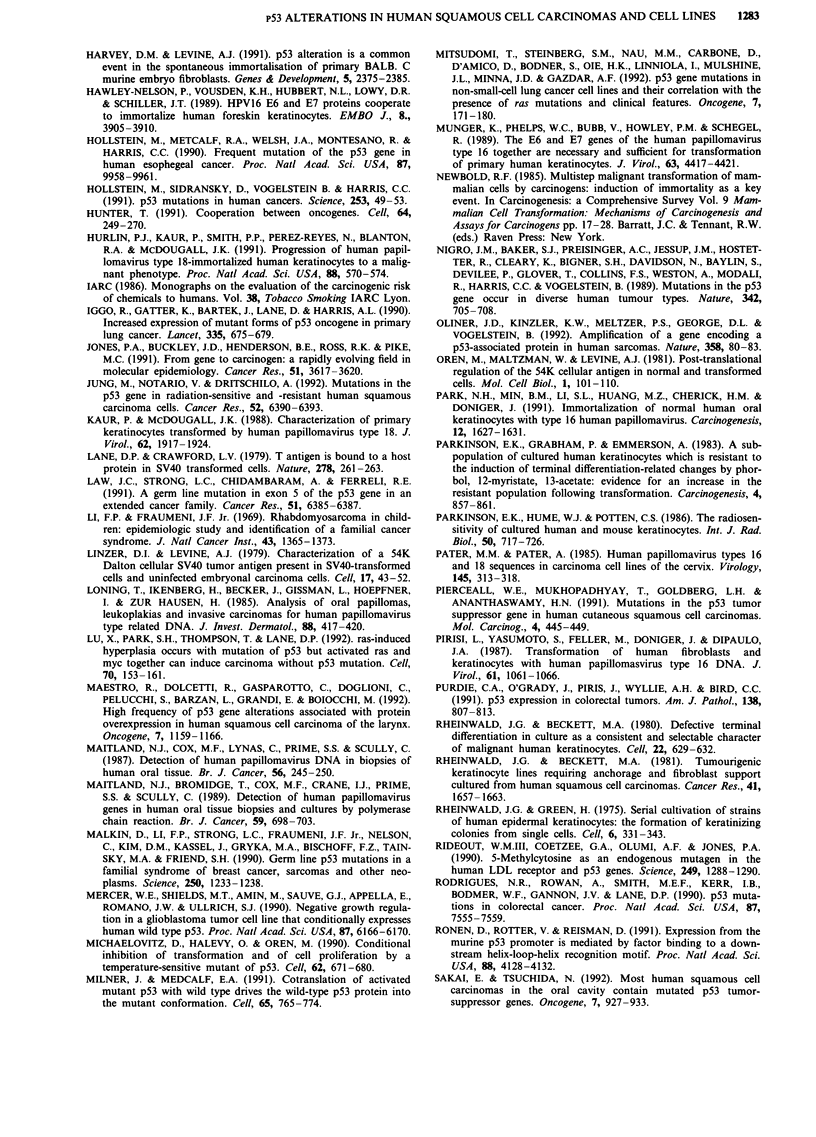

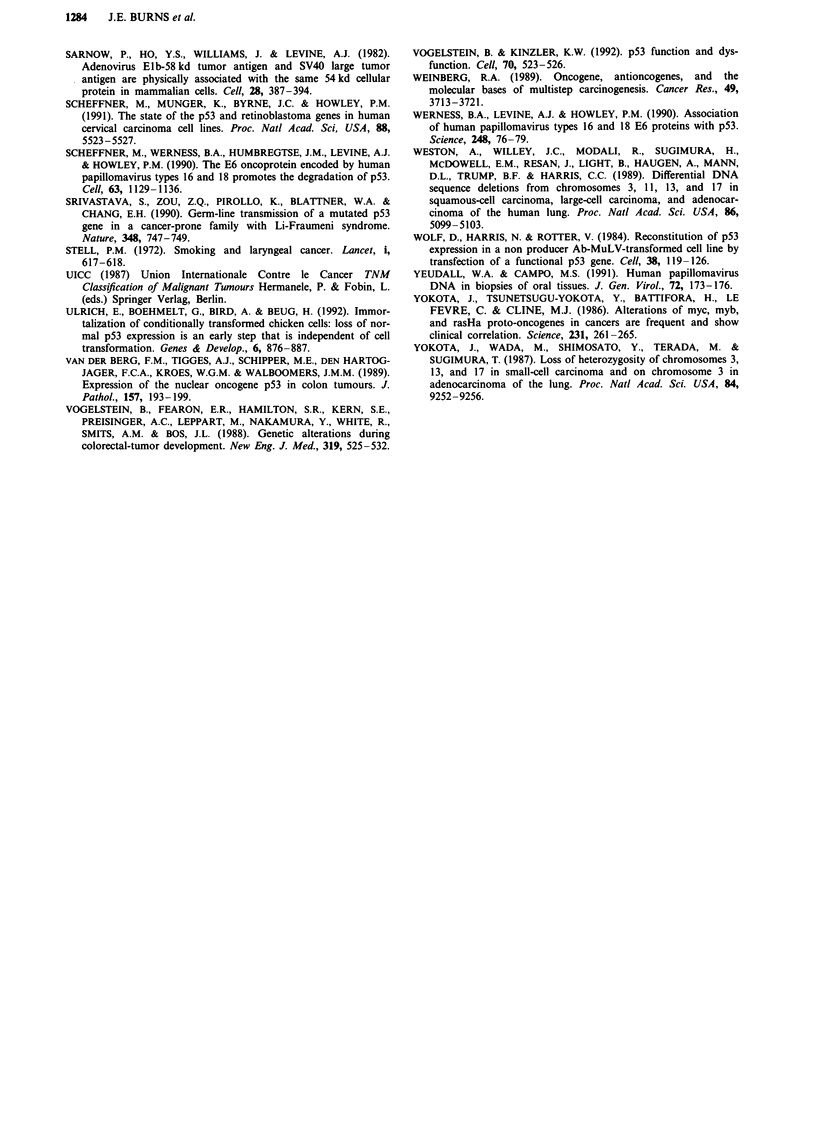


## References

[OCR_01168] Baker S. J., Fearon E. R., Nigro J. M., Hamilton S. R., Preisinger A. C., Jessup J. M., vanTuinen P., Ledbetter D. H., Barker D. F., Nakamura Y. (1989). Chromosome 17 deletions and p53 gene mutations in colorectal carcinomas.. Science.

[OCR_01173] Baker S. J., Markowitz S., Fearon E. R., Willson J. K., Vogelstein B. (1990). Suppression of human colorectal carcinoma cell growth by wild-type p53.. Science.

[OCR_01178] Banks L., Matlashewski G., Crawford L. (1986). Isolation of human-p53-specific monoclonal antibodies and their use in the studies of human p53 expression.. Eur J Biochem.

[OCR_01188] Bartek J., Iggo R., Gannon J., Lane D. P. (1990). Genetic and immunochemical analysis of mutant p53 in human breast cancer cell lines.. Oncogene.

[OCR_01195] Bennett W. P., Hollstein M. C., He A., Zhu S. M., Resau J. H., Trump B. F., Metcalf R. A., Welsh J. A., Midgley C., Lane D. P. (1991). Archival analysis of p53 genetic and protein alterations in Chinese esophageal cancer.. Oncogene.

[OCR_01200] Bischoff F. Z., Strong L. C., Yim S. O., Pratt D. R., Siciliano M. J., Giovanella B. C., Tainsky M. A. (1991). Tumorigenic transformation of spontaneously immortalized fibroblasts from patients with a familial cancer syndrome.. Oncogene.

[OCR_01205] Bischoff F. Z., Yim S. O., Pathak S., Grant G., Siciliano M. J., Giovanella B. C., Strong L. C., Tainsky M. A. (1990). Spontaneous abnormalities in normal fibroblasts from patients with Li-Fraumeni cancer syndrome: aneuploidy and immortalization.. Cancer Res.

[OCR_01212] Brachman D. G., Graves D., Vokes E., Beckett M., Haraf D., Montag A., Dunphy E., Mick R., Yandell D., Weichselbaum R. R. (1992). Occurrence of p53 gene deletions and human papilloma virus infection in human head and neck cancer.. Cancer Res.

[OCR_01219] Brash D. E., Rudolph J. A., Simon J. A., Lin A., McKenna G. J., Baden H. P., Halperin A. J., Pontén J. (1991). A role for sunlight in skin cancer: UV-induced p53 mutations in squamous cell carcinoma.. Proc Natl Acad Sci U S A.

[OCR_01184] Bártek J., Bártková J., Vojtesek B., Stasková Z., Rejthar A., Kovarík J., Lane D. P. (1990). Patterns of expression of the p53 tumour suppressor in human breast tissues and tumours in situ and in vitro.. Int J Cancer.

[OCR_01224] Caron de Fromentel C., Soussi T. (1992). TP53 tumor suppressor gene: a model for investigating human mutagenesis.. Genes Chromosomes Cancer.

[OCR_01231] Casey G., Lo-Hsueh M., Lopez M. E., Vogelstein B., Stanbridge E. J. (1991). Growth suppression of human breast cancer cells by the introduction of a wild-type p53 gene.. Oncogene.

[OCR_01235] Cattoretti G., Rilke F., Andreola S., D'Amato L., Delia D. (1988). P53 expression in breast cancer.. Int J Cancer.

[OCR_01240] Chen P. L., Chen Y. M., Bookstein R., Lee W. H. (1990). Genetic mechanisms of tumor suppression by the human p53 gene.. Science.

[OCR_01245] Chen Y. M., Chen P. L., Arnaiz N., Goodrich D., Lee W. H. (1991). Expression of wild-type p53 in human A673 cells suppresses tumorigenicity but not growth rate.. Oncogene.

[OCR_01251] Cheng J., Yee J. K., Yeargin J., Friedmann T., Haas M. (1992). Suppression of acute lymphoblastic leukemia by the human wild-type p53 gene.. Cancer Res.

[OCR_01259] Chiba I., Takahashi T., Nau M. M., D'Amico D., Curiel D. T., Mitsudomi T., Buchhagen D. L., Carbone D., Piantadosi S., Koga H. (1990). Mutations in the p53 gene are frequent in primary, resected non-small cell lung cancer. Lung Cancer Study Group.. Oncogene.

[OCR_01264] Crook T., Tidy J. A., Vousden K. H. (1991). Degradation of p53 can be targeted by HPV E6 sequences distinct from those required for p53 binding and trans-activation.. Cell.

[OCR_01268] Crook T., Wrede D., Tidy J., Scholefield J., Crawford L., Vousden K. H. (1991). Status of c-myc, p53 and retinoblastoma genes in human papillomavirus positive and negative squamous cell carcinomas of the anus.. Oncogene.

[OCR_01273] Diller L., Kassel J., Nelson C. E., Gryka M. A., Litwak G., Gebhardt M., Bressac B., Ozturk M., Baker S. J., Vogelstein B. (1990). p53 functions as a cell cycle control protein in osteosarcomas.. Mol Cell Biol.

[OCR_01280] Donehower L. A., Harvey M., Slagle B. L., McArthur M. J., Montgomery C. A., Butel J. S., Bradley A. (1992). Mice deficient for p53 are developmentally normal but susceptible to spontaneous tumours.. Nature.

[OCR_01286] Easty D. M., Easty G. C., Carter R. L., Monaghan P., Butler L. J. (1981). Ten human carcinoma cell lines derived from squamous carcinomas of the head and neck.. Br J Cancer.

[OCR_01292] Fearon E. R., Hamilton S. R., Vogelstein B. (1987). Clonal analysis of human colorectal tumors.. Science.

[OCR_01298] Field J. K., Spandidos D. A., Malliri A., Gosney J. R., Yiagnisis M., Stell P. M. (1991). Elevated P53 expression correlates with a history of heavy smoking in squamous cell carcinoma of the head and neck.. Br J Cancer.

[OCR_01302] Finlay C. A., Hinds P. W., Levine A. J. (1989). The p53 proto-oncogene can act as a suppressor of transformation.. Cell.

[OCR_01307] Finlay C. A., Hinds P. W., Tan T. H., Eliyahu D., Oren M., Levine A. J. (1988). Activating mutations for transformation by p53 produce a gene product that forms an hsc70-p53 complex with an altered half-life.. Mol Cell Biol.

[OCR_01313] Gordon A. J., Burns P. A., Fix D. F., Yatagai F., Allen F. L., Horsfall M. J., Halliday J. A., Gray J., Bernelot-Moens C., Glickman B. W. (1988). Missense mutation in the lacI gene of Escherichia coli. Inferences on the structure of the repressor protein.. J Mol Biol.

[OCR_01320] Green H. (1978). Cyclic AMP in relation to proliferation of the epidermal cell: a new view.. Cell.

[OCR_01324] Gusterson B. A., Anbazhagan R., Warren W., Midgely C., Lane D. P., O'Hare M., Stamps A., Carter R., Jayatilake H. (1991). Expression of p53 in premalignant and malignant squamous epithelium.. Oncogene.

[OCR_01330] Halevy O., Michalovitz D., Oren M. (1990). Different tumor-derived p53 mutants exhibit distinct biological activities.. Science.

[OCR_01337] Harvey D. M., Levine A. J. (1991). p53 alteration is a common event in the spontaneous immortalization of primary BALB/c murine embryo fibroblasts.. Genes Dev.

[OCR_01341] Hawley-Nelson P., Vousden K. H., Hubbert N. L., Lowy D. R., Schiller J. T. (1989). HPV16 E6 and E7 proteins cooperate to immortalize human foreskin keratinocytes.. EMBO J.

[OCR_01347] Hollstein M. C., Metcalf R. A., Welsh J. A., Montesano R., Harris C. C. (1990). Frequent mutation of the p53 gene in human esophageal cancer.. Proc Natl Acad Sci U S A.

[OCR_01353] Hollstein M., Sidransky D., Vogelstein B., Harris C. C. (1991). p53 mutations in human cancers.. Science.

[OCR_01357] Hunter T. (1991). Cooperation between oncogenes.. Cell.

[OCR_01361] Hurlin P. J., Kaur P., Smith P. P., Perez-Reyes N., Blanton R. A., McDougall J. K. (1991). Progression of human papillomavirus type 18-immortalized human keratinocytes to a malignant phenotype.. Proc Natl Acad Sci U S A.

[OCR_01370] Iggo R., Gatter K., Bartek J., Lane D., Harris A. L. (1990). Increased expression of mutant forms of p53 oncogene in primary lung cancer.. Lancet.

[OCR_01375] Jones P. A., Buckley J. D., Henderson B. E., Ross R. K., Pike M. C. (1991). From gene to carcinogen: a rapidly evolving field in molecular epidemiology.. Cancer Res.

[OCR_01380] Jung M., Notario V., Dritschilo A. (1992). Mutations in the p53 gene in radiation-sensitive and -resistant human squamous carcinoma cells.. Cancer Res.

[OCR_01385] Kaur P., McDougall J. K. (1988). Characterization of primary human keratinocytes transformed by human papillomavirus type 18.. J Virol.

[OCR_01390] Lane D. P., Crawford L. V. (1979). T antigen is bound to a host protein in SV40-transformed cells.. Nature.

[OCR_01394] Law J. C., Strong L. C., Chidambaram A., Ferrell R. E. (1991). A germ line mutation in exon 5 of the p53 gene in an extended cancer family.. Cancer Res.

[OCR_01399] Li F. P., Fraumeni J. F. (1969). Rhabdomyosarcoma in children: epidemiologic study and identification of a familial cancer syndrome.. J Natl Cancer Inst.

[OCR_01404] Linzer D. I., Levine A. J. (1979). Characterization of a 54K dalton cellular SV40 tumor antigen present in SV40-transformed cells and uninfected embryonal carcinoma cells.. Cell.

[OCR_01414] Lu X., Park S. H., Thompson T. C., Lane D. P. (1992). Ras-induced hyperplasia occurs with mutation of p53, but activated ras and myc together can induce carcinoma without p53 mutation.. Cell.

[OCR_01408] Löning T., Ikenberg H., Becker J., Gissmann L., Hoepfer I., zur Hausen H. (1985). Analysis of oral papillomas, leukoplakias, and invasive carcinomas for human papillomavirus type related DNA.. J Invest Dermatol.

[OCR_01420] Maestro R., Dolcetti R., Gasparotto D., Doglioni C., Pelucchi S., Barzan L., Grandi E., Boiocchi M. (1992). High frequency of p53 gene alterations associated with protein overexpression in human squamous cell carcinoma of the larynx.. Oncogene.

[OCR_01432] Maitland N. J., Bromidge T., Cox M. F., Crane I. J., Prime S. S., Scully C. (1989). Detection of human papillomavirus genes in human oral tissue biopsies and cultures by polymerase chain reaction.. Br J Cancer.

[OCR_01427] Maitland N. J., Cox M. F., Lynas C., Prime S. S., Meanwell C. A., Scully C. (1987). Detection of human papillomavirus DNA in biopsies of human oral tissue.. Br J Cancer.

[OCR_01441] Malkin D., Li F. P., Strong L. C., Fraumeni J. F., Nelson C. E., Kim D. H., Kassel J., Gryka M. A., Bischoff F. Z., Tainsky M. A. (1990). Germ line p53 mutations in a familial syndrome of breast cancer, sarcomas, and other neoplasms.. Science.

[OCR_01445] Mercer W. E., Shields M. T., Amin M., Sauve G. J., Appella E., Romano J. W., Ullrich S. J. (1990). Negative growth regulation in a glioblastoma tumor cell line that conditionally expresses human wild-type p53.. Proc Natl Acad Sci U S A.

[OCR_01450] Michalovitz D., Halevy O., Oren M. (1990). Conditional inhibition of transformation and of cell proliferation by a temperature-sensitive mutant of p53.. Cell.

[OCR_01455] Milner J., Medcalf E. A. (1991). Cotranslation of activated mutant p53 with wild type drives the wild-type p53 protein into the mutant conformation.. Cell.

[OCR_01460] Mitsudomi T., Steinberg S. M., Nau M. M., Carbone D., D'Amico D., Bodner S., Oie H. K., Linnoila R. I., Mulshine J. L., Minna J. D. (1992). p53 gene mutations in non-small-cell lung cancer cell lines and their correlation with the presence of ras mutations and clinical features.. Oncogene.

[OCR_01468] Münger K., Phelps W. C., Bubb V., Howley P. M., Schlegel R. (1989). The E6 and E7 genes of the human papillomavirus type 16 together are necessary and sufficient for transformation of primary human keratinocytes.. J Virol.

[OCR_01474] Newbold R. F. (1985). Multistep malignant transformation of mammalian cells by carcinogens: induction of immortality as a key event.. Carcinog Compr Surv.

[OCR_01484] Nigro J. M., Baker S. J., Preisinger A. C., Jessup J. M., Hostetter R., Cleary K., Bigner S. H., Davidson N., Baylin S., Devilee P. (1989). Mutations in the p53 gene occur in diverse human tumour types.. Nature.

[OCR_01490] Oliner J. D., Kinzler K. W., Meltzer P. S., George D. L., Vogelstein B. (1992). Amplification of a gene encoding a p53-associated protein in human sarcomas.. Nature.

[OCR_01494] Oren M., Maltzman W., Levine A. J. (1981). Post-translational regulation of the 54K cellular tumor antigen in normal and transformed cells.. Mol Cell Biol.

[OCR_01499] Park N. H., Min B. M., Li S. L., Huang M. Z., Cherick H. M., Doniger J. (1991). Immortalization of normal human oral keratinocytes with type 16 human papillomavirus.. Carcinogenesis.

[OCR_01505] Parkinson E. K., Grabham P., Emmerson A. (1983). A subpopulation of cultured human keratinocytes which is resistant to the induction of terminal differentiation-related changes by phorbol, 12-myristate, 13-acetate: evidence for an increase in the resistant population following transformation.. Carcinogenesis.

[OCR_01513] Parkinson E. K., Hume W. J., Potten C. S. (1986). The radiosensitivity of cultured human and mouse keratinocytes.. Int J Radiat Biol Relat Stud Phys Chem Med.

[OCR_01518] Pater M. M., Pater A. (1985). Human papillomavirus types 16 and 18 sequences in carcinoma cell lines of the cervix.. Virology.

[OCR_01523] Pierceall W. E., Mukhopadhyay T., Goldberg L. H., Ananthaswamy H. N. (1991). Mutations in the p53 tumor suppressor gene in human cutaneous squamous cell carcinomas.. Mol Carcinog.

[OCR_01529] Pirisi L., Yasumoto S., Feller M., Doniger J., DiPaolo J. A. (1987). Transformation of human fibroblasts and keratinocytes with human papillomavirus type 16 DNA.. J Virol.

[OCR_01535] Purdie C. A., O'Grady J., Piris J., Wyllie A. H., Bird C. C. (1991). p53 expression in colorectal tumors.. Am J Pathol.

[OCR_01540] Rheinwald J. G., Beckett M. A. (1980). Defective terminal differentiation in culture as a consistent and selectable character of malignant human keratinocytes.. Cell.

[OCR_01545] Rheinwald J. G., Beckett M. A. (1981). Tumorigenic keratinocyte lines requiring anchorage and fibroblast support cultured from human squamous cell carcinomas.. Cancer Res.

[OCR_01551] Rheinwald J. G., Green H. (1975). Serial cultivation of strains of human epidermal keratinocytes: the formation of keratinizing colonies from single cells.. Cell.

[OCR_01556] Rideout W. M., Coetzee G. A., Olumi A. F., Jones P. A. (1990). 5-Methylcytosine as an endogenous mutagen in the human LDL receptor and p53 genes.. Science.

[OCR_01560] Rodrigues N. R., Rowan A., Smith M. E., Kerr I. B., Bodmer W. F., Gannon J. V., Lane D. P. (1990). p53 mutations in colorectal cancer.. Proc Natl Acad Sci U S A.

[OCR_01566] Ronen D., Rotter V., Reisman D. (1991). Expression from the murine p53 promoter is mediated by factor binding to a downstream helix-loop-helix recognition motif.. Proc Natl Acad Sci U S A.

[OCR_01572] Sakai E., Tsuchida N. (1992). Most human squamous cell carcinomas in the oral cavity contain mutated p53 tumor-suppressor genes.. Oncogene.

[OCR_01579] Sarnow P., Ho Y. S., Williams J., Levine A. J. (1982). Adenovirus E1b-58kd tumor antigen and SV40 large tumor antigen are physically associated with the same 54 kd cellular protein in transformed cells.. Cell.

[OCR_01585] Scheffner M., Münger K., Byrne J. C., Howley P. M. (1991). The state of the p53 and retinoblastoma genes in human cervical carcinoma cell lines.. Proc Natl Acad Sci U S A.

[OCR_01591] Scheffner M., Werness B. A., Huibregtse J. M., Levine A. J., Howley P. M. (1990). The E6 oncoprotein encoded by human papillomavirus types 16 and 18 promotes the degradation of p53.. Cell.

[OCR_01597] Srivastava S., Zou Z. Q., Pirollo K., Blattner W., Chang E. H. (1990). Germ-line transmission of a mutated p53 gene in a cancer-prone family with Li-Fraumeni syndrome.. Nature.

[OCR_01603] Stell P. M. (1972). Smoking and laryngeal cancer.. Lancet.

[OCR_01612] Ulrich E., Boehmelt G., Bird A., Beug H. (1992). Immortalization of conditionally transformed chicken cells: loss of normal p53 expression is an early step that is independent of cell transformation.. Genes Dev.

[OCR_01624] Vogelstein B., Fearon E. R., Hamilton S. R., Kern S. E., Preisinger A. C., Leppert M., Nakamura Y., White R., Smits A. M., Bos J. L. (1988). Genetic alterations during colorectal-tumor development.. N Engl J Med.

[OCR_01630] Vogelstein B., Kinzler K. W. (1992). p53 function and dysfunction.. Cell.

[OCR_01634] Weinberg R. A. (1989). Oncogenes, antioncogenes, and the molecular bases of multistep carcinogenesis.. Cancer Res.

[OCR_01639] Werness B. A., Levine A. J., Howley P. M. (1990). Association of human papillomavirus types 16 and 18 E6 proteins with p53.. Science.

[OCR_01644] Weston A., Willey J. C., Modali R., Sugimura H., McDowell E. M., Resau J., Light B., Haugen A., Mann D. L., Trump B. F. (1989). Differential DNA sequence deletions from chromosomes 3, 11, 13, and 17 in squamous-cell carcinoma, large-cell carcinoma, and adenocarcinoma of the human lung.. Proc Natl Acad Sci U S A.

[OCR_01653] Wolf D., Harris N., Rotter V. (1984). Reconstitution of p53 expression in a nonproducer Ab-MuLV-transformed cell line by transfection of a functional p53 gene.. Cell.

[OCR_01658] Yeudall W. A., Campo M. S. (1991). Human papillomavirus DNA in biopsies of oral tissues.. J Gen Virol.

[OCR_01662] Yokota J., Tsunetsugu-Yokota Y., Battifora H., Le Fevre C., Cline M. J. (1986). Alterations of myc, myb, and rasHa proto-oncogenes in cancers are frequent and show clinical correlation.. Science.

[OCR_01668] Yokota J., Wada M., Shimosato Y., Terada M., Sugimura T. (1987). Loss of heterozygosity on chromosomes 3, 13, and 17 in small-cell carcinoma and on chromosome 3 in adenocarcinoma of the lung.. Proc Natl Acad Sci U S A.

[OCR_01620] van den Berg F. M., Tigges A. J., Schipper M. E., den Hartog-Jager F. C., Kroes W. G., Walboomers J. M. (1989). Expression of the nuclear oncogene p53 in colon tumours.. J Pathol.

